# Comparison of the Physical Activity and Sedentary Behaviour Assessment Questionnaire and the Short-Form International Physical Activity Questionnaire: An Analysis of Health Survey for England Data

**DOI:** 10.1371/journal.pone.0151647

**Published:** 2016-03-18

**Authors:** Shaun Scholes, Sally Bridges, Linda Ng Fat, Jennifer S. Mindell

**Affiliations:** 1 Health and Social Surveys Research Group, Research Department of Epidemiology and Public Health, University College London, 1-19 Torrington Place, London, WC1E 6BT, United Kingdom; 2 NatCen Social Research, 35 Northampton Square, London, EC1V 0AX, United Kingdom; University Of São Paulo, BRAZIL

## Abstract

**Background:**

The Physical Activity and Sedentary Behaviour Assessment Questionnaire (PASBAQ), used within the Health Survey for England (HSE) at 5-yearly intervals, is not included annually due to funding and interview-length constraints. Policy-makers and data-users are keen to consider shorter instruments such as the Short-form International Physical Activity Questionnaire (IPAQ) for the annual survey. Both questionnaires were administered in HSE 2012, enabling comparative assessment in a random sample of 1252 adults.

**Methods:**

Relative agreement using prevalence-adjusted bias-adjusted Kappa (PABAK) statistics was estimated for: sufficient aerobic activity (moderate-to-vigorous physical activity [MVPA] ≥150minutes/week); inactivity (MVPA<30minutes/week); and excessive sitting (≥540minutes/weekday). Cross-sectional associations with health outcomes were compared across tertiles of MVPA and tertiles of sitting time using logistic regression with tests for linear trend.

**Results:**

Compared with PASBAQ data, IPAQ-assessed estimates of sufficient aerobic activity and inactivity were higher and lower, respectively; estimates of excessive sitting were higher. Demographic patterns in prevalence were similar. Agreement using PABAK statistics was fair-to-moderate for sufficient aerobic activity (0.32–0.49), moderate-to-substantial for inactivity (0.42–0.74), and moderate-to-substantial for excessive sitting (0.49–0.75). As with the PASBAQ, IPAQ-assessed MVPA and sitting each showed graded associations with mental well-being (women: *P* for trend = 0.003 and 0.004, respectively) and obesity (women: *P* for trend = 0.007 and 0.014, respectively).

**Conclusions:**

Capturing habitual physical activity and sedentary behaviour through brief questionnaires is complex. Differences in prevalence estimates can reflect differences in questionnaire structure and content rather than differences in reported behaviour. Treating all IPAQ-assessed walking as moderate-intensity contributed to the differences in prevalence estimates. PASBAQ data will be used for population surveillance every 4 to 5 years. The current version of the Short-form IPAQ was included in HSE 2013–14 to enable more frequent assessment of physical activity and sedentary behaviour; a modified version with different item-ordering and additional questions on walking-pace and effort was included in HSE 2015.

## Introduction

Both physical inactivity and sedentary behaviour (characterised by activities involving prolonged sitting) are independent risk factors for chronic diseases such as cardiovascular disease (CVD) and obesity [1,2]. Clustering of risk factors including physical inactivity as well as poor diet and smoking has also been associated with ill-health [3]. Inactivity costs the United Kingdom National Health Service (NHS) £1.1billion directly [4], with indirect costs to society bringing this cost to a total of £8.2billion [5,6].

Feasibility and costs are important considerations for choosing self-report or device-based methods to assess physical activity or sedentary behaviour. Despite decreasing costs for device-based measures, reported methods remain less expensive than device-based methods, especially for large studies [7]. Data collected from physical activity questionnaires within large population-based health examination surveys is used to monitor secular trends, quantitatively examine associations between physical activity and health [8,9], and to enable statistical adjustment for physical activity as a potential mediator or moderator in many associations of interest in epidemiologic research [10,11]. Assessing the volume and intensity of habitual physical activity and sedentary behaviour through the administration of questionnaires that are short enough to meet space- and time-constraints is a complex task [12–14]. The Health Survey for England (HSE) is a nationally representative, population-based survey that collects multiple-domain physical activity data along with a wealth of socio-demographic variables, objective measures of height and weight, and blood pressure measurements. Within the HSE, data on leisure-time physical activity and sedentary behaviour is collected using the Physical Activity and Sedentary Behaviour Assessment Questionnaire (PASBAQ). PASBAQ data have been extensively used to monitor adherence to UK physical activity recommendations [15–18] and for other epidemiologic research [9,19–21]. Strengths of the PASBAQ include its measurement of each component of physical activity: frequency, duration, and intensity within multiple domains (e.g., domestic activity, sports and exercise, and walking). Convergent validity of the PASBAQ has been indicated by its ability to clearly grade anthropometric and biological outcomes, such as body mass index, resting pulse rate, and HDL cholesterol, according to physical activity levels [9]. PASBAQ-assessed sedentary behaviour has also been shown to be consistently associated with cardio-metabolic outcomes such as body mass index and waist circumference [21]. Comparisons with accelerometer data also showed that the PASBAQ is a useful and valid instrument for ranking individuals according to levels of physical activity and sedentary behaviour [22,23]. Limitations of the PASBAQ are similar to those of other long, detailed physical activity questionnaires collected within large national health examination surveys that gather data on multiple topics, namely its expensive costs and high respondent burden (it takes on average 12 minutes to complete).

Due to funding and interview-length constraints, the PASBAQ cannot be included each year. This prevents the inclusion of physical activity and sedentary behaviour data in analyses in the survey years when it is not collected [24]. Both policy-makers and data-users are keen to include more frequently some assessment of physical activity and sedentary behaviour as major risk factors for cardiovascular disease and some cancers. Physical activity was the most frequently mentioned topic for data that was required annually in a recent consultation of users of HSE data by the Health and Social Care Information Centre [25]. NatCen Social Research was therefore keen to examine the usefulness of including a short physical activity questionnaire in the annual HSE, and so funded the costs of including a shorter instrument in the HSE from 2012 to 2015. One candidate for inclusion was the Short-form International Physical Activity Questionnaire (IPAQ) which covers the frequency and duration of vigorous, moderate, and walking activities over the last 7 days, as well as a single-item question on weekday sitting. Strengths of the Short-form IPAQ include its relative cost-effectiveness, partly through its lower demands on participants (it takes on average two minutes to complete). It has been widely researched and has improved the standardisation of physical activity and sedentary behaviour measurement for international comparability [26,27]. It has been deemed acceptable for use in physical activity research and surveillance activities, showing good reliability, acceptable criterion validity, and reasonable classification accuracy compared with accelerometer data [26,28–30] and physical activity related energy-expenditure through the doubly-labelled water method [31]. The limitations of the Short-form IPAQ are similar to those of other short, brief physical activity questionnaires, namely that it is generic and focuses on total activity (i.e., does not measure separate domains of activity other than walking). Participants are required to make their own judgements about the intensity of their activities across all contexts simultaneously, leading to possible overestimation of the volume of time spent doing activities of sufficient intensity for health benefits due to “spill-over effects” where participants report light-intensity activities as moderate-intensity, and report moderate-intensity activities as vigorous. The placement of vigorous- before moderate-intensity items has also been identified as a possible source of double-counting of activity [32,33].

Questionnaire design is an iterative process. It is well recognised that efforts should continue in developing high-quality self-report measures of physical activity and sedentary behaviour, including a need for shorter instruments for use in large national studies where questionnaire space is limited [34]. In England, PASBAQ data collected within the HSE is used to monitor changes over time in adherence to UK physical activity recommendations [35]. To ensure consistency in the time-series, PASBAQ data will continue to be used for population surveillance but only at 4- to 5-yearly intervals.

Both the PASBAQ and Short-form IPAQ were administered to the same set of participants in HSE 2012 allowing us an opportunity to undertake a comparative assessment to examine the usefulness of including a shorter instrument in future annual rounds of the HSE to complement occasional use of the PASBAQ. Given this intended application, the most important correlation is that between the two self-report instruments, and not their level of agreement with device-based methods.

The aim of this study was to compare the physical activity and sedentary behaviour data obtained from the two instruments. More specifically, our objectives were to: (1) compare PASBAQ- and IPAQ-assessed prevalence estimates of sufficient aerobic activity, inactivity, and excessive sitting, and examine the similarities in patterning across population subgroups; (2) quantify their level of agreement in identifying the same set of participants as being above or below commonly used duration thresholds; and (3) compare their cross-sectional associations with a range of physical health and mental health variables. These objectives are consistent with the use of physical activity questionnaires in large-scale surveys to produce estimates for population subgroups rather than to detect individual behaviour change as in the clinical setting [11].

## Materials and Methods

### Study design and analytical sample

HSE data is used to monitor progress on many national health objectives, including physical activity in 1998, 2003, 2004, 2006, 2008, and 2012 [34]. Details about the HSE sample design are described elsewhere [36]. Briefly, the HSE annually draws a nationally-representative sample of persons aged ≥16years living in private households in England using multistage stratified probability sampling with postcode sectors as the primary sampling unit and the Postcode Address File as the sampling frame for households. Fieldwork is conducted continuously through the year.

Trained interviewers measured participants’ height and weight and assessed their demographic characteristics, self-reported health, and health behaviours using computer-assisted personal interviewing. Following this, participants had a visit from a trained nurse. The response rate for both the main interview and nurse-visit (within co-operating households) was 56%. The PASBAQ was administered during the main interview; additionally, for participants in the fourth quarter of fieldwork (October 2012-February 2013), the Short-form IPAQ was administered during the nurse-visit, which occurs a few days to a few weeks later. Our analytical sample consisted of 1252 participants aged ≥16years who completed both questionnaires. The distribution of the analytical sample for the number of days between the main interview and nurse-visit was as follows: 1–13 days (28%), 14–27 days (28%), 28–41 days (20%), and 42 days or more (24%).

### Assessment of physical activity and sedentary behaviour

#### Physical Activity and Sedentary Behaviour Assessment Questionnaire (PASBAQ)

Detailed information on the PASBAQ is available elsewhere [37]. Briefly, questions included the frequency (number of days in the last four weeks) and duration (of an average episode) of participation in four domains of leisure-time physical activity: (1) “light” (e.g., general tidying) and “heavy” (e.g., spring cleaning) domestic activity; (2) “light” and “heavy” manual work/gardening/do-it-yourself activity; (3) walking (with no distinction between walking for leisure or commuting); and (4) sports/exercise. Intensity of walking was assessed by asking participants if their usual walking-pace was slow, average, fairly brisk, or fast. Participants aged ≥65years were also asked whether the effort of walking for ≥10minutes was usually enough to make them “*breathe faster*, *feel warmer or sweat*” [37]. Intensity of sports/exercise was determined by the nature of the activity as indexed in the metabolic equivalent (MET) compendium of Ainsworth and colleagues [38,39] and a follow-up question on whether the activity had made the participant “*out of breath or sweaty*”. Sedentary behaviour during leisure-time, on weekdays and at weekend days, was assessed using a set of questions on the usual amount of time spent in: (1) television viewing (including digital video discs (DVDs)) and (2) any other (non-television-viewing) sitting, including reading and computer use.

#### Occupational physical activity

As part of the main interview, participants aged 16–74 engaged in employment were asked on how many workdays, in the last four weeks, their work included: (1) climbing of stairs/ladders, or (2) lifting, carrying or moving heavy loads, followed by a question about the average time spent on that activity on a typical workday. Including these activities for participants in specific occupations allows an assessment of overall volumes of moderate-to-vigorous physical activity (MVPA), and as such is taken into account in the estimation of adherence to current UK physical activity recommendations [37].

#### Short-form International Physical Activity Questionnaire (IPAQ)

Participants reported the frequency and duration of: (1) vigorous (examples given included heavy lifting, fast bicycling), (2) moderate (carrying light loads and bicycling at a regular pace), and (3) walking activities, as well as the average time spent sitting on a weekday, including sitting at work, during the last seven days.

Self-report instruments such as the PASBAQ and IPAQ prompt participants to report activities lasting at least 10 minutes, reflecting global recommendations on physical activity for health which state that aerobic activity should be performed in bouts of at least 10 minutes duration [40]. Further information on the structure and content of both questionnaires as used in HSE 2012 is shown in Table 1.

**Table 1:** Structure and content of the physical activity questionnaires (PASBAQ and Short-form IPAQ) used in HSE 2012.

Structure and content	PASBAQ	Short-form IPAQ
**Data collection phase**	Interview	Nurse visit
**Time to administer**	~12 minutes	~2 minutes
**Level of detail**	Detailed module	7 questions
**Recall period**	Last 28 days	Last 7 days
**Minimum duration of activity bouts included**	10 minutes	10 minutes
**Domains of PA assessed**	Housework; manual/gardening/do-it-yourself; walking; sports/exercise	Walking
**List of specific activities**	Showcards and activities read out by a respondent and coded in the interview from a list in the interview programme	Examples
**Definition for vigorous-intensity physical activity**	Subset of sports/exercise (as indexed by MET compendium) and follow-up question on whether activity had made participants “out-of-breath or sweaty”	Frequency and duration of: “activities that take hard physical effort and make you breathe much harder than normal. Examples include heavy lifting, digging, aerobics, fast bicycling.”
**Definition for moderate-intensity physical activity**	(1) “Heavy” housework (e.g., digging, refitting a kitchen/bathroom)	(1) Frequency and duration of: “activities that take moderate physical effort and make you breathe somewhat harder than normal. Examples include carrying light loads, bicycling at a regular pace, doubles tennis.”
	(2) “Heavy” manual (e.g., moving heavy furniture, cleaning windows)	
	(3) Walking of at least moderate-intensity (see below)	(2) All walking (see below)
	(4) Subset of sports/exercise (as indexed by MET compendium) and follow-up question on whether activity had made participants “out-of-breath or sweaty”	
**Walking**	Includes walking to and from work, and all other walking done for recreation, sport, exercise, or leisure.	Frequency and duration of walking which “includes at work and at home, walking to travel from place to place, and any other walking that you have done solely for recreation, sport, exercise, or leisure”.
**Walking of at least moderate-intensity**	Participants who reported fairly brisk/fast-paced walking (≥16 years), and for whom the pace of walking was slow/average-paced but for whom the effort was usually enough to make them “breathe faster, feel warmer, or sweat” (aged ≥65 years)	Intensity of walking not assessed.
**Sedentary behaviour (excessive sitting)**	Non-occupational sitting: (1) Television-viewing (weekday, weekend days); (2) Non-television-viewing (weekday, weekend days)	Sitting on weekdays including “time spent at work, at home, while doing course work and during leisure time. This may include time spent sitting at a desk, visiting friends, reading, or sitting or lying down to watch television.”

IPAQ, International Physical Activity Questionnaire; MET, metabolic equivalent; MVPA, moderate-to-vigorous physical activity; PA, physical activity; PASBAQ, Physical Activity and Sedentary Behaviour Assessment Questionnaire

### Summary measures of physical activity and sedentary behaviour

Summary measures of physical activity and sedentary behaviour derived from the PASBAQ and Short-form IPAQ are outlined in Table 2. A brief description is provided here.

**Table 2:** Derivation of summary variables (PASBAQ and IPAQ) used in HSE 2012.

Variable	PASBAQ	Short-form IPAQ
**Adherence to aerobic activity recommendations (sufficiently active)**	*Primary analysis*	*Primary analysis*
	MVPA ≥150minutes/week (including moderate-intensity walking)	MVPA ≥150minutes/week (all walking included)
	*Sensitivity analysis*	*Sensitivity analysis*
	(1) MVPA ≥150minutes/week (excluding moderate-intensity walking); (2) MVPA ≥150minutes/week (excluding occupational activity)	(1) MVPA ≥150minutes/week (excluding all walking); (2) MVPA ≥150minutes/week (excluding occupational activity)
**Inactivity**	*Primary analysis*	*Primary analysis*
	MVPA <30minutes/week (including moderate-intensity walking)	MVPA <30minutes/week (all walking included)
	*Sensitivity analysis*	*Sensitivity analysis*
	MVPA <30minutes/week (excluding moderate-intensity walking)	MVPA <30minutes/week (excluding all walking)
**Excessive sedentary behaviour**	Sitting down ≥540minutes/weekday	Sitting down ≥540minutes/weekday

IPAQ, International Physical Activity Questionnaire; MVPA, moderate-to-vigorous physical activity; PASBAQ, Physical Activity and Sedentary Behaviour Assessment Questionnaire

#### Summary measures from the PASBAQ

Time spent sitting was calculated as the sum of television and non-television-viewing: sedentary behaviour (i.e., excessive sitting) was defined as spending 540 minutes or more sitting on weekdays. Time spent in moderate-intensity physical activity (MPA) was calculated as minutes per week (frequency × duration) spent in: (1) “heavy” domestic activity; (2) “heavy” manual/gardening activity; (3) moderate-intensity walking; (4) occupational activity (as described above); and (5) a subset of sports/exercise (METs: 3.0–5.9 in accordance with the Compendium of Physical Activities [38,39]). Walking of at least moderate-intensity was defined as fairly brisk or fast-paced (all participants), or walking of an average or slow pace that made participants breathe faster, feel warmer or sweat (aged ≥65years). Time spent in vigorous-intensity physical activity (VPA) was calculated as minutes per week in sports/exercises with METs ≥6.0. The average minutes/week spent in MVPA was calculated by summing time spent in MPA and in VPA, and was grouped into one of two categories (<150minutes/week or ≥150minutes/week, with time spent in VPA given twice the credit of time spent in MPA) to indicate achievement of current recommendations [15]. Participants were categorised as inactive if they spent <30minutes/week in MVPA. Sex-specific tertiles of time spent: (1) in MVPA, and (2) sitting on weekdays were calculated to categorise participants as low, medium, or high for subsequent analyses of relative agreement (see below).

#### Summary measures from the Short-form IPAQ

Time spent sitting was derived from the single-item “*During the last 7 days*, *how much time did you usually spend sitting on a weekday”*. The frequency and duration of walking was assessed but not its intensity. In our primary analysis all IPAQ-assessed walking was assumed to be of at least moderate-intensity. This assumption was made for two reasons. First, our assumption was in line with a number of previous studies which assumed all walking to be of at least moderate-intensity by assigning MET values of 3.3 or 4.0 in accordance with the IPAQ scoring protocol [41] and the Compendium of Physical Activities respectively [38,39]. Secondly, despite their differential treatment of walking, both instruments may nevertheless identify the same population subgroups at the lower tail of the MVPA distribution that are most at risk of ill-health. Each summary measure of physical activity and sedentary behaviour was derived in the same way as described above for the PASBAQ. Estimates of MVPA ≥3600 minutes/week were truncated at 3600minutes/week.

### Demographics and health variables

Single measurements of height and weight were taken using standard protocols. Body mass index (BMI) was computed as weight in kilogrammes (kg) divided by height in metres squared (m^2^), and was grouped into three categories: normal weight (18.5–24.9kg/m^2^), overweight (25.0–29.9kg/m^2^), and obese (≥30.0kg/m^2^). Participants with BMI <18.5kg/m^2^ were excluded from BMI-specific analyses due to small numbers. Annual household income was established using a card showing 30 bands (from less than £520 to £150,000 or more). Equivalised household income was calculated (annual household income divided by the McClemens scoring system) and grouped into tertiles. Three blood pressure readings were taken (Omron HEM 207 monitor, Omron, Japan). Resting heart rate (RHR), a marker of physical fitness [42], was calculated in beats-per-minute based on the average of the second and third reading. Sex-specific tertiles of resting heart rate were created to categorise participants as low, medium, or high.

Positive mental well-being was measured by the Warwick-Edinburgh Mental Well-Being Scale (WEMWBS) [43]. Responses to 14 statements (each ranging from 1 to 5) were aggregated to form the Well-being Index, with higher scores indicating higher positive well-being. Participants having a score below the 10^th^ percentile were classified as having a low WEMWBS score. Participants were classified as having CVD if they reported any of the following physical conditions or illnesses, lasting or expected to last 12 months or more: angina, heart attack, stroke, heart murmur, or irregular heart rhythm. Smoking status categories were current smoker, ex-regular smoker, and never been a regular smoker. Responses to questions on alcohol consumption on the heaviest drinking day in the last 7 days were used to categorise participants as below / more than twice in excess of the NHS recommended daily limits (thus >6units for women; >8units for men). Hypertension was defined as systolic blood pressure of ≥140mmHg and/or diastolic blood pressure of ≥90mmHg and/or current use of medication to lower blood pressure [44]. Total cholesterol was measured from non-fasting blood samples. Raised cholesterol was defined as total cholesterol ≥5.0mmol/l irrespective of medication use [45].

### Statistical analyses

Relationships between PASBAQ- and IPAQ-assessed MVPA and sitting time (expressed as continuous measures) were summarised using the Pearson correlation (r) and Lin’s concordance correlation coefficient (P_c_). The concordance correlation coefficient evaluates the degree to which paired data fall on the line of equality (i.e., the 45-degree line through the origin) [46,47]. Our analytical strategy focused mainly on categorical comparisons: reflecting the primary purpose of the Short-form IPAQ on the categorical reporting of levels of physical activity and sedentary behaviour [48]. Three sets of analyses were conducted to: (1) compare PASBAQ- and IPAQ-assessed prevalence estimates of sufficient aerobic activity, inactivity, and excessive sitting, and examine the similarities in patterning across population subgroups; (2) estimate the strength of relative agreement; and (3) compare similarities in cross-sectional associations with the physical health and mental health variables listed above.

#### Prevalence estimates

Analyses were run separately using each instrument to compare prevalence estimates of: (1) sufficient aerobic activity (MVPA ≥150minutes/week); (2) inactivity (MVPA <30minutes/week); and (3) excessive sedentary behaviour (sitting ≥540minutes/weekday). The threshold duration for aerobic activity was chosen to compare levels of adherence to current UK physical activity recommendations [15]. We chose a threshold duration for inactivity consistent with the lowest category of MVPA used in HSE reporting [37]; the same definition is also used by a leading UK pressure group at the forefront of a campaign aimed towards “turning the tide of inactivity” [5]. Epidemiologic evidence has not yet been sufficiently developed to define a threshold duration for health-compromising sitting time, and unlike aerobic activity, there is, as yet, no specific national guideline for sedentary behaviour for adults. Despite their differential treatment of sedentary behaviour (PASBAQ: leisure-time sitting; IPAQ: total volume of sitting), the same threshold of sitting on average ≥540minutes/weekday was used to examine the extent to which using the same threshold in different contexts captured the same group of participants. The threshold of ≥540minutes/weekday was chosen to be consistent with the highest quintile of IPAQ-assessed sitting time in the 20-country comparison of the descriptive epidemiology of sitting [27].

#### Relative agreement between instruments

The Kappa statistic was used as a measure of relative agreement between instruments that was not attributable to chance [49]. However the Kappa statistic on its own is difficult to interpret meaningfully as its magnitude is influenced by the: (1) prevalence of the attribute, and (2) bias (the extent to which the instruments disagree on the proportion of positive / negative cases) [50,51]. We computed the prevalence-adjusted bias-adjusted Kappa (PABAK) statistic, with the accompanying prevalence- and bias-indices (PI and BI respectively), to provide an indication of the likely effects of prevalence and bias on the unadjusted Kappa [52]. The strength of agreement for 2×2 tables using both Kappa and PABAK statistics was interpreted according to Landis and Koch’s classification: <0.20 (“slight” agreement); 0.21–0.40 (“fair”); 0.41–0.60 (“moderate”); 0.61–0.80 (“substantial”); and 0.81+ (“almost-perfect”) [53]. The quadratic weighted Kappa statistic for 3×3 tables was used to compare tertiles of MVPA and tertiles of sitting time [54]. To examine whether agreement differed across population subgroups analyses were stratified by gender, age-group (16–44, 45–64, ≥65), BMI status (normal/overweight/obese), tertiles of income, and tertiles of resting heart rate.

#### Similarities in cross-sectional associations with physical health and mental health variables

Analyses were run separately using each instrument to compare cross-sectional associations with other health variables. Sex-specific prevalence estimates of obesity, having a low WEMWBS score, CVD, current cigarette smoking, drinking more than twice in excess of recommended daily alcohol limits on the heaviest drinking day in the last 7 days, hypertension, and raised cholesterol were computed according to the PASBAQ- and IPAQ-based tertiles of time spent in MVPA and the tertiles of time spent sitting. Logistic regression was performed with the health outcome as the dependent variable and the three categories of MVPA and of sitting as the independent variable. First, the tertiles were entered in the models using two indicator variables with the group representing the lowest tertile as reference. An adjusted Wald test was used for testing overall significance. Secondly, the tertiles were entered as one continuous variable with the *P*-value used as a test for linear trend. These analyses were conducted on age-standardised data, using the 2012 English household population as standard, to take into account the differences in age profile across the categories of MVPA and of sitting. We assessed difference in linear trend across the instruments using the significance level of the coefficient for an interaction term (i.e., PASBAQ × IPAQ), which was added to the model including both instruments as a main effect.

#### Sensitivity analyses

Selection bias potentially results in estimates for subgroups not being representative of the true levels in the entire study population [51]. The HSE 2012 sample consisted of two groups: PASBAQ with IPAQ (the analytical sample), and PASBAQ without IPAQ. Potential differences in the amount of time spent in MVPA and in the amount of time spent sitting between the two groups across confounding covariates (not including physical activity or sitting time) was examined by propensity score analysis [55]. Logistic regression was used to estimate the propensity score: the dependent variable being sample type (0 = PASBAQ without IPAQ; 1 = PASBAQ with IPAQ), with sex, age, region, number of adults and children in the household, BMI, marital status, income tertiles, presence of CVD, smoking status, adherence to NHS recommended daily alcohol limits, National Statistics Socio-Economic Classification, and the main interview non-response weight as independent variables. Quintiles of the estimated propensity score were created, with the first quintile representing the highest probability of having PASBAQ and IPAQ data, and the fifth quintile representing the lowest. Within each quintile, participant characteristics were balanced across the two groups. Within each quintile, mean scores of MPVA and of sitting time (using the longer-form PASBAQ) were compared across the two sample groups to assess whether the difference in the amount of time spent in MVPA and in the amount of time spent sitting on weekdays were independent of the response propensity.

PASBAQ questions on usual walking-pace and effort (aged ≥65years) are used to distinguish between light- and moderate-intensity walking: with only the latter counting towards adherence to UK physical activity recommendations. Earlier versions of the Short-form IPAQ included questions about walking-pace. These have since been removed given their minimal contribution to estimates of reliability and validity with accelerometer data used as the criterion [26]. Domain-specific analyses have identified walking as the largest contributor to overall volumes of physical activity [56] and, more specifically, to volumes of MVPA [17,57]. In our main analysis, calculations for IPAQ-assessed MVPA assumed all walking to be of at least moderate-intensity. As a sensitivity analysis, we estimated the level of relative agreement excluding all walking from the time spent in MVPA.

Consistent with HSE reporting, a subset of occupational physical activity for participants in a specific set of occupations [37] was accounted for in the assessment of MVPA. The questionnaire section on occupational activity, administered as part of the main interview, is lengthy and detailed, and as with the PASBAQ, cannot be included each year. Hence, as a further sensitivity analysis, we repeated our primary analysis excluding occupational physical activity from MVPA.

Data management was performed using SPSS version 20.0 (SPSS Inc., Chicago, Illinois, US), and analysis was conducted using Stata version 13.1 (StataCorp LP, College Station, Texas, US) accounting for the complex sample design. Tests of statistical significance were based on two-sided probability (*P*<0.05).

#### Ethics statement

Each sampled address for the HSE is sent an advance letter which introduces the survey and states that an interviewer would be calling to seek permission to interview. A leaflet is also enclosed providing general information about the survey and some of the findings from previous surveys. Individual interviews are conducted with adults who give verbal informed consent. At the end of individual interviews, participants are asked for agreement to a follow-up visit by a trained nurse. Written consent is obtained for collection of non-fasting blood samples. There is no formal record that participants have given verbal consent to the individual interview or give physical measurements that are not biological samples (e.g., height, weight, and blood pressure). It is made clear in the advance letters and information leaflets that participation in the survey is entirely voluntary, and that participants may decline to answer individual questions, withdraw or stop at any time, or refuse any particular measurement if they wish to do so. Interviewers and nurses will often repeat this information in their introductions and when they are setting up appointments, and throughout the interview as necessary. Indeed, many individuals do refuse to participate in the survey; others may refuse individual questions, decline to continue part way through an interview or refuse physical measurements. It is also standard practice to conduct interviews and nurse visits some time after an appointment has been made so that individuals have a chance to reflect on their agreement before the appointment takes place. The procedures used in the HSE to obtain informed consent are very closely scrutinised by a National Health Service (NHS) ethics committee each year. Information leaflets and both the content and wording of questionnaires are also carefully reviewed by the ethics committees. Ethical approval for HSE 2012 was obtained from the Oxfordshire A Research Ethics Committee (reference 10/H0604/56). This study is a secondary analysis of previously collected data and so additional ethical approval was not required.

## Results

### Characteristics of the sample

Of the 8291 adults interviewed in HSE 2012, 8173 completed the PASBAQ. 2325 individuals were interviewed in the fourth quarter, and, of these, 1252 co-operated with the nurse-visit and completed the Short-form IPAQ. Statistically significant differences in demographic characteristics were examined by comparing the 1252 participants in the analytical sample (PASBAQ with IPAQ) with the subset of the main HSE 2012 sample that comprised 6921 participants with PASBAQ but without IPAQ data (Table 3). The analytical sample was older on average than the sample with PASBAQ but no IPAQ data (mean age 49.1 and 46.0 years respectively; *P*<0.001) and contained more married than single people (*P* = 0.024), but did not differ with regard to other socio-demographic characteristics such as gender, socioeconomic status, and self-reported health conditions. 63.3% of participants in the analytical sample met current physical activity recommendations (MVPA ≥150minutes/week) according to the PASBAQ, compared with 61.2% in the full sample (data not shown).

**Table 3:** Characteristics of participants in the Health Survey for England 2012 by sample type (PASBAQ without IPAQ and PASBAQ with IPAQ).

Characteristic	PASBAQ without IPAQ (*n* = 6921)		PASBAQ with IPAQ (*n* = 1251)		*P*-value^a^
	%	Mean (SD)	%	Mean (SD)	
**Men**	49.0		48.0		0.413
**Age, years**		46.0 (18.9)		49.1 (18.1)	<0.001
**Age-group**:					
16–44	50.0		41.4		<0.001
45–64	30.9		37.2		
65+	19.2		21.4		
**Marital status**:					
Married/cohabiting	61.5		63.6		0.024
Single	24.5		20.1		
Other	14.0		16.3		
Missing	0.0		-		
**NS-SEC**:					
Managerial and professional	32.0		33.7		0.243
Intermediate	23.4		24.5		
Routine and manual	36.4		36.0		
Other	6.4		4.5		
Missing	1.8		1.3		
**BMI, kg/m**^**2**^		27.1 (5.3)		27.3 (5.3)	0.477
**BMI category**:					
Under 18.5	1.5		1.0		0.444
18.5 and below 25	29.9		34.3		
25 and below 30	30.6		33.7		
30 and below 40	18.0		21.9		
Over 40	2.0		2.2		
Missing	17.9		7.0		
**Resting heart rate, bpm**		52.8 (13.8)		53.4 (14.2)	0.328
**Tertiles of resting heart rate**^b^:					
Lowest	16.8		30.2		0.276
Middle	17.6		29.3		
Highest	12.6		24.6		
Missing	53.1		15.9		
**Tertiles of equivalised household income**:					
Highest	27.2		29.7		0.138
Middle	26.0		29.8		
Lowest	25.4		22.6		
Missing	21.4		17.8		
**MVPA ≥150 minutes/week**	60.8		63.3		0.181
**Sitting ≥540 minutes/weekday**	9.1		8.7		0.652
**Current smoker**	20.3		17.3		0.114
**Excessive alcohol consumption**	16.2		17.9		0.623
**Reported CVD**	10.5		11.0		0.627
**Hypertension**	24.8		26.3		0.417
**Raised cholesterol**	60.7		63.4		0.238
**Bottom 10% of WEMWBS**	10.2		10.5		0.839

The Physical Activity and Sedentary Behaviour Assessment Questionnaire (PASBAQ) was administered in the interview; the Short-form International Physical Activity Questionnaire (IPAQ) was administered in the nurse visit. Sample counts un-weighted. Estimates were weighted by the interview-weight variable. Column percentages may not sum to 100 because of rounding error.

BMI, body mass index; bpm, beats per minute; CVD, cardiovascular disease; MVPA, moderate-to-vigorous physical activity; NS-SEC, National Statistics Socio-Economic Classification; SD, standard deviation; WEMWBS, Warwick-Edinburgh Mental Well-Being Scale.

^a^ *P*-values calculated by means of the χ^2^ test (categorical variables) or *t*-test (continuous variables).

^b^ Sex-specific cut-points for defining the heart rate tertiles: Men (24.0–49.5; 50.0–60.5; 61.0–119.0 bpm); Women (1.0–43.5; 44.0–56.5; 57.0–168.0 bpm).

**Fig 1:**
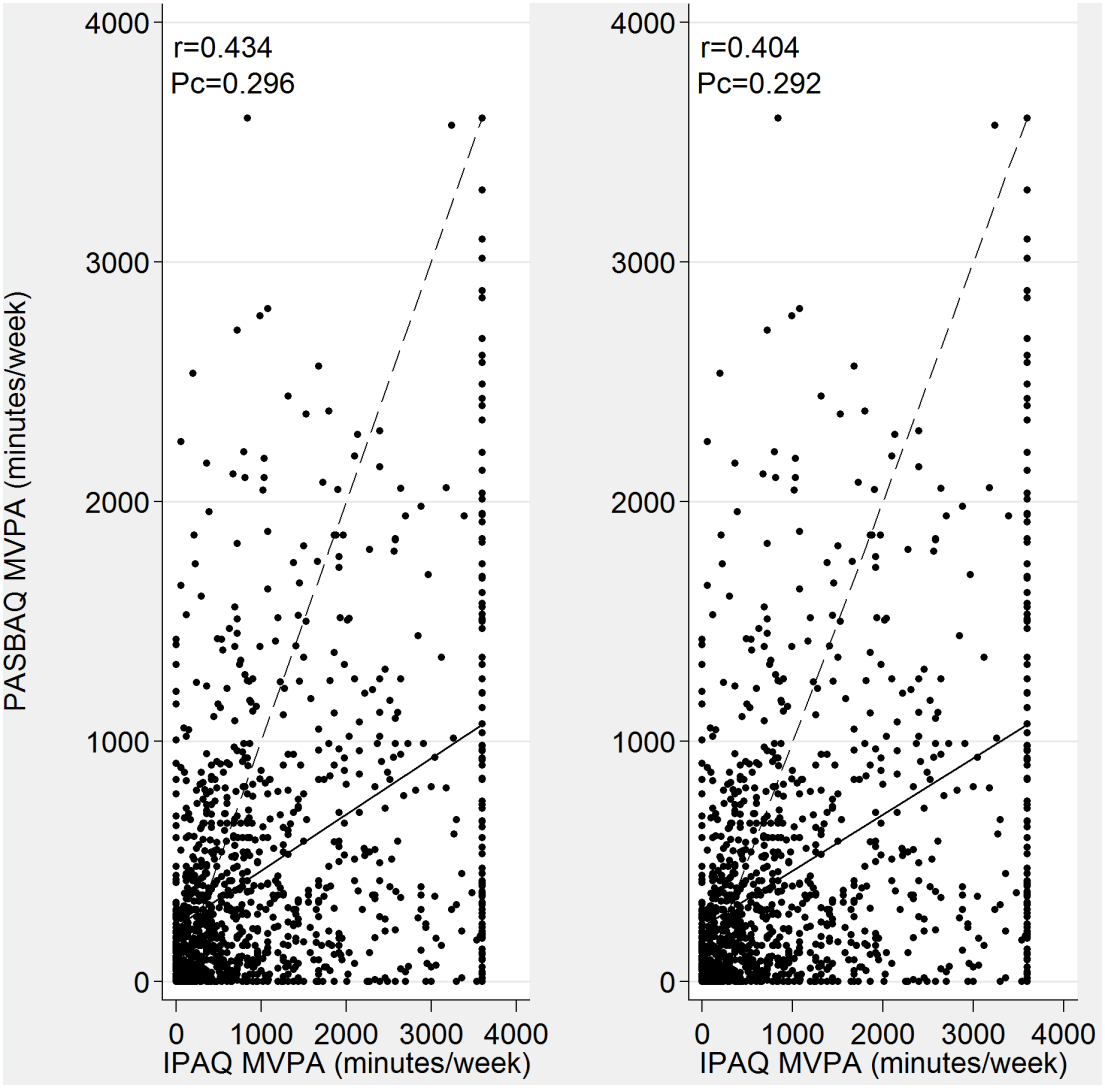
Relationships between PASBAQ and IPAQ assessed MVPA for men (left panel) and women (right panel). Solid line represents the fitted linear regression line; dotted line represents the 45 degree line of equality (indicating perfect agreement). Pearson (r) and concordance (P_c_) correlation coefficients shown.

**Fig 2:**
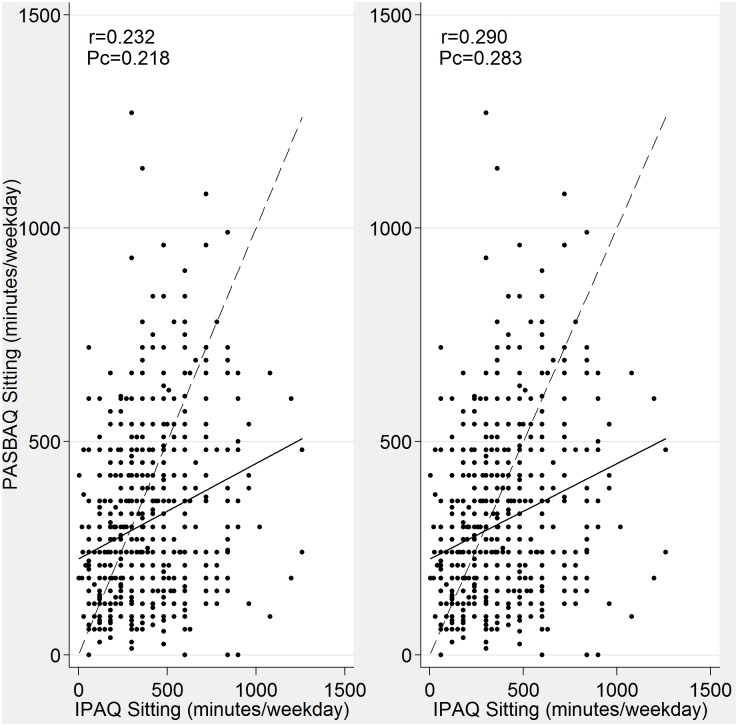
Relationships between PASBAQ and IPAQ assessed time spent sitting on weekdays for men (left panel) and women (right panel). Solid line represents the fitted linear regression line; dotted line represents the 45 degree line of equality (indicating perfect agreement). Pearson (r) and concordance (P_c_) correlation coefficients shown.

Scatterplots of the continuous measures showed the familiar pattern of increased scatter as time spent in MVPA and time spent sitting on weekdays increased (Figs 1 and 2). Pearson’s correlation was modest for MVPA (r = 0.434 for men and r = 0.404 for women) but poor for sitting time. Concordance correlation coefficients were poor (P_c_<0.30), reflecting the large divergence of the data points from the fitted regression line, and the divergence of the fitted line from the 45-degree line of equality through the origin.

### Prevalence estimates of sufficient aerobic activity, inactivity, and excessive sitting

**Fig 3:**
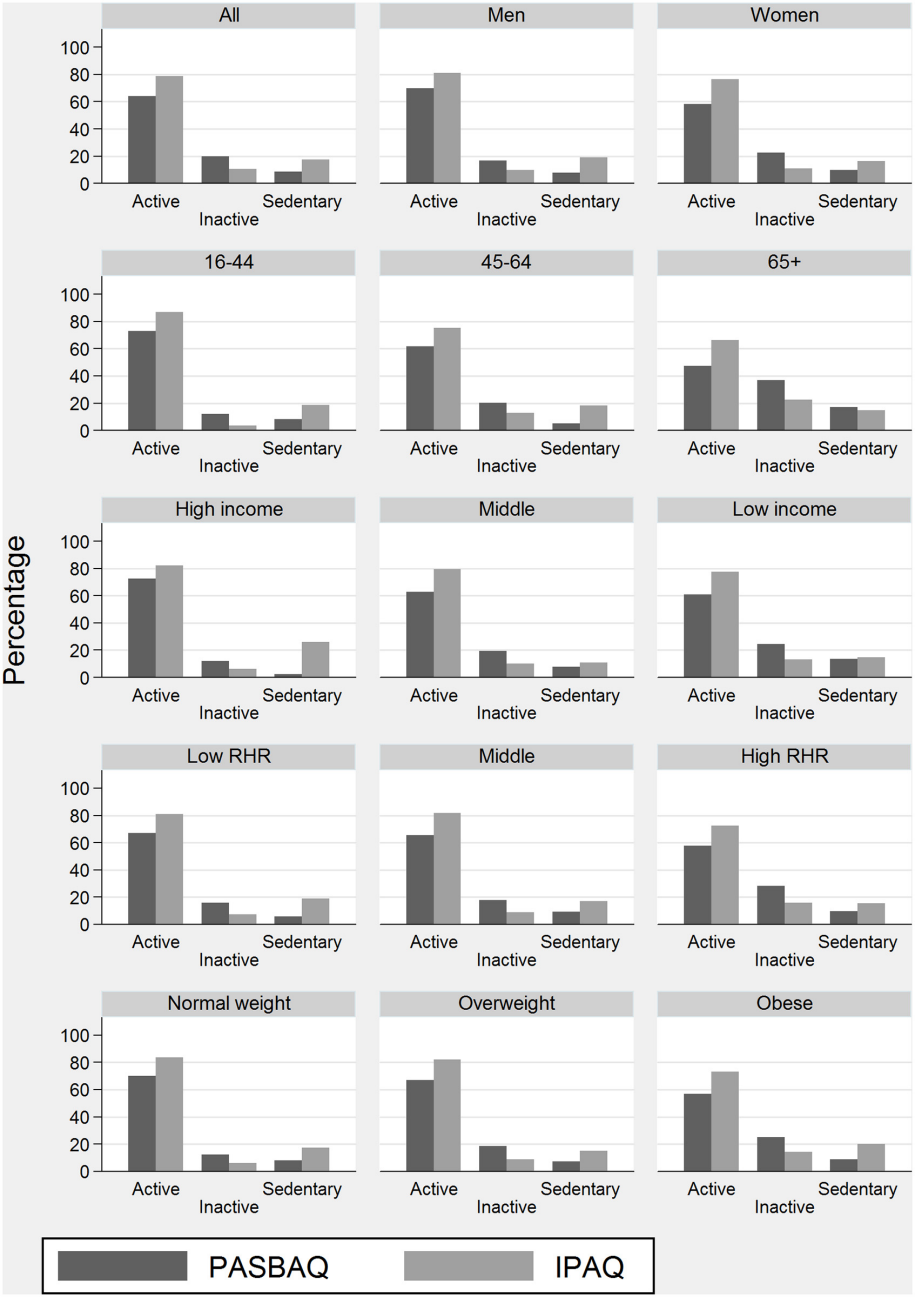
Prevalence of sufficient aerobic activity, inactivity, and excessive sitting according to the PASBAQ and Short-form IPAQ. Proportion of participants categorised as: (1) sufficiently aerobically active (moderate-to-vigorous physical activity [MVPA] ≥150minutes/week), 2) inactive (MVPA <30minutes/week), and 3) sedentary (sitting ≥540minutes/weekday) according to the Physical Activity and Sedentary Behaviour Assessment Questionnaire (PASBAQ) and Short-form International Physical Activity Questionnaire (IPAQ) across groups stratified by gender, age-group, income, resting pulse rate (RHR), and BMI category.

Fig 3 compares the PASBAQ- and Short-form IPAQ-assessed prevalence estimates of sufficient aerobic activity, inactivity, and excessive sitting. Higher levels of activity were demonstrated across both questionnaires for men than for women, for those aged 16–44 than for older adults, for those in the highest than in the lowest income group, and for those with normal weight than those classed as overweight or obese. IPAQ-based estimates of sufficient aerobic activity were higher and estimates of inactivity were lower than the PASBAQ for all participants combined, and for groups stratified by gender, age, income, resting heart rate, and BMI. IPAQ-based estimates of sufficient aerobic activity ranged from 9.6% to 18.9% percentage points higher than the PASBAQ. IPAQ-based estimates of excessive sitting were higher than the PASBAQ amongst most subgroups, with the gap between estimates most pronounced for participants aged 16–44 (18.5% *vs*. 8.1%), 45–64 (18.3% *vs*. 5.0%), and in the highest income category (25.8% *vs*. 2.5%).

### Relative agreement between questionnaires

Table 4 shows the Kappa and prevalence-adjusted bias-adjusted Kappa (PABAK) statistics, with the accompanying prevalence- and bias-indices (PI and BI respectively), for the three dichotomous PASBAQ and IPAQ classifications arranged in 2×2 tables. PABAK values were higher than the Kappa across all three estimates, reflecting high values for the prevalence index. The strength of agreement for PABAK statistics according to Landis and Koch’s classification was fair-to-moderate for sufficient aerobic activity (ranging from 0.32 to 0.49), moderate-to-substantial for inactivity (0.42 to 0.74), and moderate-to-substantial for excessive sitting (0.49 to 0.75).

**Table 4:** Kappa statistic and 95% CI, and the prevalence-adjusted bias-adjusted Kappa (PABAK) statistic for PASBAQ- and IPAQ-based estimates of sufficient aerobic activity, inactivity, and excessive sitting.

	Sufficient activity^a^					Inactivity^a^					Excessive sitting				
	(MVPA ≥150minutes/week)					(MVPA <30minutes/week)					(≥540minutes/weekday)				
	Kappa (95% CI)	K_max_	PABAK	PI	BI	Kappa (95% CI)	K_max_	PABAK	PI	BI	Kappa (95% CI)	K_max_	PABAK	PI	BI
**All**	0.31 (0.26–0.37)	0.65	0.42	-0.43	0.15	0.26 (0.20–0.33)	0.64	0.62	0.70	-0.09	0.15 (0.08–0.22)	0.61	0.60	0.74	0.09
**Sex**:															
Men	0.32 (0.23–0.41)	0.70	0.49	-0.51	0.11	0.32 (0.22–0.42)	0.71	0.68	0.73	-0.07	0.11 (0.02–0.20)	0.50	0.58	0.74	0.12
Women	0.30 (0.23–0.37)	0.60	0.36	-0.35	0.18	0.21 (0.13–0.30)	0.59	0.55	0.66	-0.12	0.20 (0.10–0.30)	0.71	0.63	0.74	0.07
**Age-group**:															
16–44	0.23 (0.13–0.33)	0.58	0.49	-0.60	0.14	0.03 (0.00–0.11)	0.42	0.72	0.85	-0.08	0.10 (0.00–0.21)	0.57	0.57	0.73	0.10
45–64	0.32 (0.23–0.41)	0.69	0.40	-0.37	0.14	0.27 (0.16–0.39)	0.72	0.60	0.67	-0.08	0.06 (0.00–0.15)	0.39	0.59	0.77	0.13
65+	0.33 (0.23–0.42)	0.63	0.32	-0.14	0.19	0.32 (0.21–0.42)	0.66	0.42	0.40	-0.15	0.43 (0.30–0.57)	0.97	0.71	0.69	-0.01
**BMI group**:															
Normal	0.30 (0.20–0.41)	0.64	0.49	-0.53	0.13	0.24 (0.10–0.38)	0.63	0.74	0.82	-0.06	0.01 (0.00–0.12)	0.60	0.55	0.75	0.09
Overweight	0.26 (0.16–0.35)	0.60	0.42	-0.49	0.15	0.17 (0.06–0.27)	0.60	0.60	0.72	-0.10	0.24 (0.10–0.37)	0.60	0.69	0.78	0.08
Obese	0.27 (0.16–0.38)	0.65	0.32	-0.30	0.16	0.21 (0.08–0.33)	0.66	0.49	0.60	-0.11	0.07 (0.00–0.19)	0.54	0.53	0.71	0.12
**Income**:															
Highest	0.18 (0.07–0.29)	0.73	0.42	-0.55	0.10	0.16 (0.03–0.29)	0.64	0.72	0.82	-0.06	0.05 (0.00–0.11)	0.14	0.49	0.71	0.23
Middle	0.30 (0.20–0.40)	0.62	0.41	-0.42	0.16	0.19 (0.07–0.31)	0.64	0.59	0.71	-0.09	0.26 (0.08–0.43)	0.82	0.75	0.81	0.03
Lowest	0.41 (0.31–0.52)	0.62	0.48	-0.38	0.17	0.41 (0.29–0.53)	0.64	0.63	0.62	-0.11	0.22 (0.07–0.37)	0.92	0.63	0.72	0.02
**Heart rate**:															
Lowest	0.26 (0.15–0.37)	0.65	0.41	-0.48	0.14	0.14 (0.01–0.27)	0.59	0.65	0.77	-0.09	0.12 (0.00–0.23)	0.38	0.61	0.76	0.14
Middle	0.29 (0.18–0.39)	0.60	0.43	-0.47	0.16	0.24 (0.12–0.37)	0.63	0.64	0.73	-0.09	0.19 (0.04–0.34)	0.68	0.62	0.73	0.08
Highest	0.40 (0.30–0.49)	0.68	0.43	-0.30	0.15	0.32 (0.21–0.43)	0.64	0.52	0.56	-0.12	0.24 (0.09–0.38)	0.68	0.67	0.76	0.07

BI, bias-index; BMI, body mass index; CI, confidence interval; IPAQ, Short-form International Physical Activity Questionnaire; κ_max_, maximum attainable value of the Kappa statistic; MVPA, moderate-to-vigorous physical activity; PABAK, prevalence-adjusted bias-adjusted Kappa statistic; PASBAQ, physical activity and sedentary behaviour questionnaire; PI, prevalence-index.

Bias-index denotes the difference between disagreements; Prevalence-index denotes the difference between agreements on the positive and negative classification.

^a^ PASBAQ-defined sufficient aerobic activity and inactivity included walking of at least moderate-intensity only; IPAQ-defined sufficient aerobic activity and inactivity included all walking as the intensity of walking was not assessed.

Unadjusted Kappa statistics for sufficient aerobic activity and for inactivity varied across population subgroups, being highest for groups with the lowest volumes of MVPA: participants aged ≥65years, in the lowest income group, and in the group with the highest values of resting heart rate. Agreement across BMI categories failed to show any consistent pattern. PABAK statistics for sufficient aerobic activity and for inactivity showed a different pattern in some instances, with values being highest for men, participants aged 16–44, and in the group with normal weight. Relative agreement for excessive sitting also varied across subgroups, with values being highest for participants aged ≥65years and in the group with the highest values of resting heart rate.

Quadratic weighted Kappa statistics across the PASBAQ- and IPAQ-based tertiles were higher for MVPA (κ = 0.31 to 0.42) than for time spent sitting (κ = 0.12 to 0.35) (Table 5). Differences in the strength of relative agreement across subgroups were more marked for sedentary behaviour than for MVPA, with the weighted Kappa statistics for time spent sitting being highest for women, for participants aged ≥65years, for those in the lowest income group, and for participants with the highest values of resting heart rate.

**Table 5:** Percentage agreement and weighted Kappa coefficients for PASBAQ- and IPAQ-based tertiles of MVPA and of sitting.

	N	Tertiles of time spent in MVPA	Tertiles of time spent sitting
		% agreement, weighted Kappa (95% CI)	
**All**	1252	48.4%, 0.39 (0.34–0.43)	44.2%, 0.22 (0.16–0.27)
**Sex**:			
Men	548	49.6%, 0.41 (0.32–0.46)	43.0%, 0.15 (0.07–0.24)
Women	704	47.1%, 0.37 (0.28–0.43)	45.3%, 0.27 (0.21–0.34)
**Age-group**:			
16–44	427	48.9%, 0.37 (0.30–0.47)	45.1%, 0.19 (0.12–0.31)
45–64	477	47.8%, 0.38 (0.32–0.44)	40.8%, 0.13 (0.01–0.23)
65+	348	48.0%, 0.35 (0.25–0.42)	48.1%, 0.35 (0.24–0.41)
**BMI group**:			
Normal	407	47.9%, 0.40 (0.31–0.48)	43.0%, 0.21 (0.12–0.36)
Overweight	435	47.2%, 0.31 (0.21–0.39)	43.9%, 0.19 (0.08–0.36)
Obese	302	46.8%, 0.37 (0.28–0.48)	46.3%, 0.23 (0.12–0.34)
**Income**:			
Highest	366	41.8%, 0.33 (0.28–0.43)	38.6%, 0.12 (0.05–0.18)
Middle	361	51.4%, 0.39 (0.28–0.47)	47.9%, 0.30 (0.19–0.41)
Lowest	307	47.8%, 0.41 (0.35–0.56)	50.2%, 0.35 (0.27–0.46)
**Heart rate**:			
Lowest	352	47.7%, 0.39 (0.27–0.47)	42.5%, 0.12 (0.06–0.21)
Middle	358	52.9%, 0.42 (0.33–0.49)	41.3%, 0.22 (0.12–0.34)
Highest	350	47.2%, 0.41 (0.32–0.48)	45.8%, 0.30 (0.20–0.37)

BMI, body mass index; CI, confidence interval; IPAQ, Short-form International Physical Activity Questionnaire; MVPA, moderate-to-vigorous physical activity; PASBAQ, Physical Activity and Sedentary Behaviour Assessment Questionnaire.

Counts are unweighted: estimates weighted.

### Consistency in associations with physical health and mental health variables

**Fig 4:**
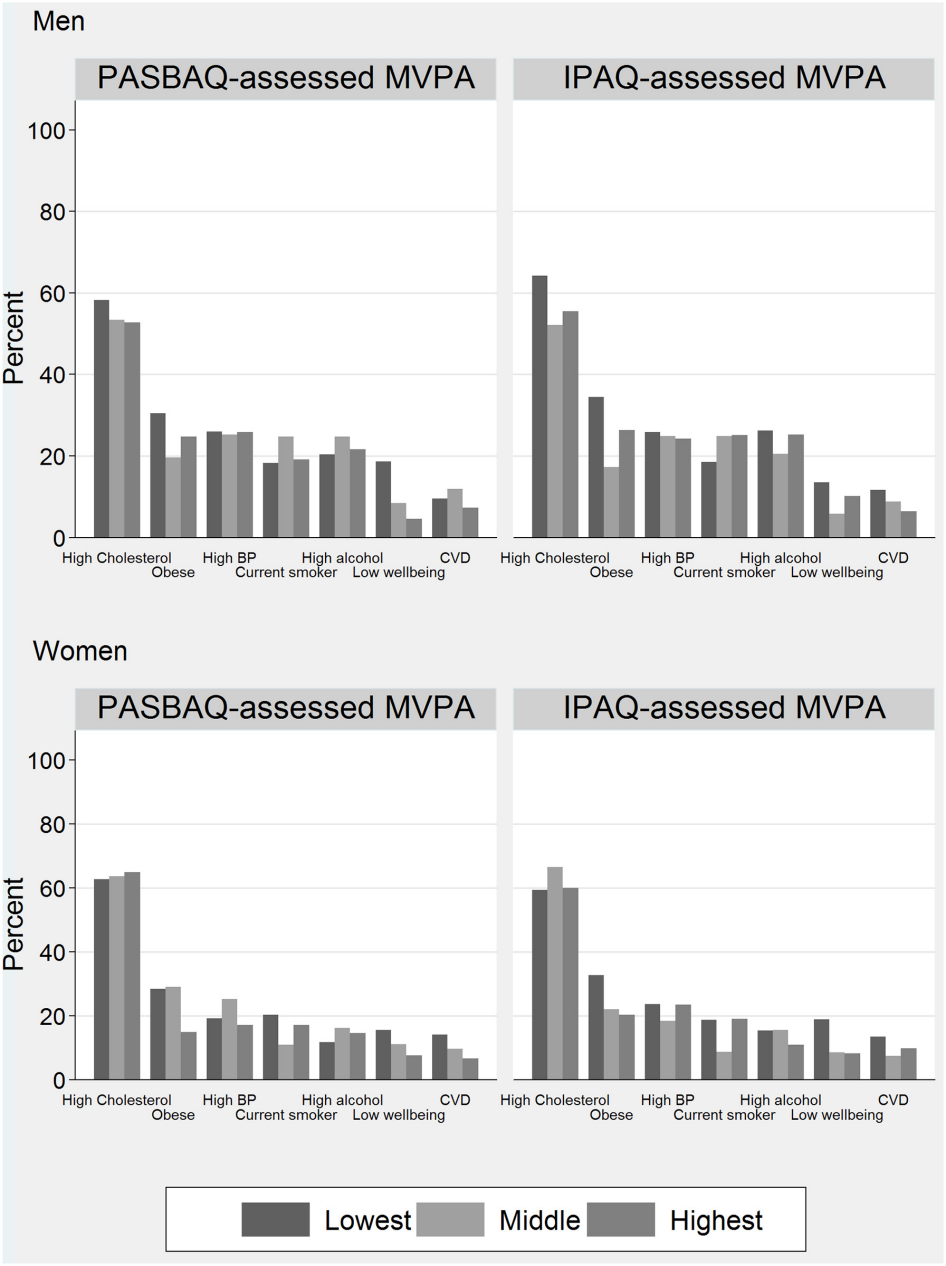
Prevalence of health outcomes according to the PASBAQ- and Short-form IPAQ-assessed tertiles of time spent in MVPA for men (top panel) and women (lower panel). Proportion of participants categorised with physical health and mental health outcomes according to the Physical Activity and Sedentary Behaviour Assessment Questionnaire (PASBAQ) and Short-form International Physical Activity Questionnaire (IPAQ) assessed tertiles of time spent in Moderate to Vigorous Physical Activity (MVPA).

**Fig 5:**
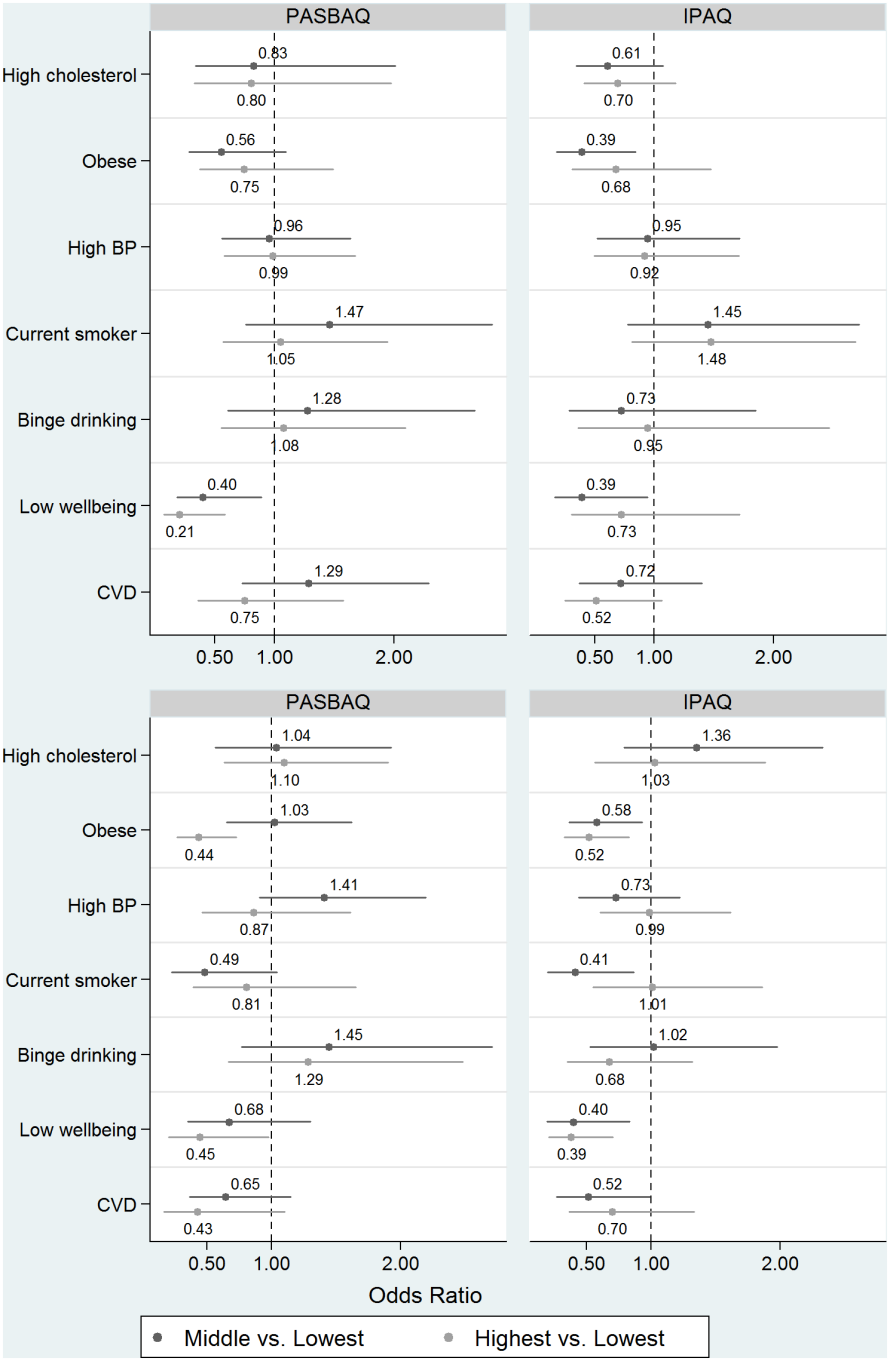
Associations of PASBAQ- and IPAQ-assessed time spent in MVPA with odds of unfavourable health outcomes for men (top panel) and women (lower panel). The odds ratios (and 95% CI) shown in Table 6 are shown in graphical form. The estimates compare participants in: (1) the middle tertile of MVPA *vs*. the lowest tertile of MVPA (dark grey), and (2) the highest tertile of MVPA *vs*. the lowest tertile of MVPA (light grey), grouped by health outcome.

Fig 4 shows the age-standardised prevalence estimates of seven physical health and mental health variables according to the PASBAQ- and IPAQ-assessed tertiles of time spent in MVPA. Table 6 shows the corresponding odds ratios with the *P*-values for group differences (adjusted Wald test) and for linear trend. P-values for the PASBAQ × IPAQ term examining the difference in linear trend across the instruments are also shown. PASBAQ data showed a number of apparent dose-response associations, some of which were seen only among men or women. Higher MVPA was associated with lower odds of having a low WEMWBS score (men: *P* for trend = 0.003; women: *P* for trend = 0.043), lower odds of being classed as obese (women: *P* for trend = 0.001), and marginally significant lower odds of reporting CVD (women: *P* for trend = 0.069). IPAQ-assessed MVPA showed similar graded associations with having a low WEMWBS score (women: *P* for trend = 0.003), obesity (women: *P* for trend = 0.007), and was marginally significant for CVD (men: *P* for trend = 0.069). Null associations across both questionnaires were found for excessive alcohol consumption, hypertension, and raised cholesterol (Fig 5). P-values for the PASBAQ × IPAQ interaction term did not reach statistical significance for any health outcome.

**Table 6:** Associations of PASBAQ- and IPAQ-assessed time spent in moderate-to-vigorous intensity physical activity with odds of unfavourable health outcomes.

Health outcomes	PASBAQ-MVPA				IPAQ-MVPA				PASBAQ and IPAQ
	Middle	Highest	*P*^a^	*P*^b^	Middle	Highest	*P*^a^	*P*^b^	*P*^c^
	Odds ratio (95% CI)				Odds ratio (95% CI)				
**Men**									
Raised cholesterol	0.83 (0.34–2.01)	0.80 (0.33–1.98)	0.889	0.637	0.61 (0.34–1.07)	0.70 (0.41–1.18)	0.191	0.252	0.063
Obese	0.56 (0.28–1.09)	0.75 (0.37–1.49)	0.230	0.485	0.39 (0.18–0.84)	0.68 (0.31–1.47)	0.036	0.395	0.813
Hypertension	0.96 (0.56–1.63)	0.99 (0.58–1.68)	0.985	0.973	0.95 (0.52–1.72)	0.92 (0.50–1.71)	0.965	0.791	0.991
Current smoker	1.47 (0.76–2.82)	1.05 (0.57–1.95)	0.434	0.998	1.45 (0.78–2.72)	1.48 (0.81–2.68)	0.399	0.206	0.620
Above alcohol limits	1.28 (0.61–2.68)	1.08 (0.55–2.09)	0.769	0.886	0.73 (0.29–1.85)	0.95 (0.36–2.46)	0.599	0.937	0.473
Low WEMWBS	0.40 (0.18–0.89)	0.21 (0.07–0.58)	0.007	0.003	0.39 (0.16–0.94)	0.73 (0.31–1.71)	0.114	0.530	0.132
Self-reported CVD	1.29 (0.73–2.29)	0.75 (0.36–1.58)	0.353	0.370	0.72 (0.37–1.40)	0.52 (0.25–1.06)	0.193	0.069	0.393
**Women**									
Raised cholesterol	1.04 (0.56–1.93)	1.10 (0.64–1.90)	0.941	0.726	1.36 (0.79–2.33)	1.03 (0.57–1.88)	0.381	0.941	0.753
Obese	1.03 (0.65–1.62)	0.44 (0.27–0.73)	0.001	0.001	0.58 (0.37–0.93)	0.52 (0.33–0.83)	0.016	0.007	0.289
Hypertension	1.41 (0.91–2.19)	0.87 (0.47–1.61)	0.134	0.602	0.73 (0.44–1.22)	0.99 (0.61–1.62)	0.394	0.984	0.839
Current smoker	0.49 (0.23–1.04)	0.81 (0.40–1.65)	0.166	0.604	0.41 (0.20–0.86)	1.01 (0.55–1.86)	0.022	0.951	0.546
Above alcohol limits	1.45 (0.77–2.71)	1.29 (0.67–2.48)	0.507	0.469	1.02 (0.53–1.98)	0.68 (0.35–1.32)	0.312	0.253	0.162
Low WEMWBS	0.68 (0.35–1.30)	0.45 (0.21–0.98)	0.132	0.043	0.40 (0.20–0.83)	0.39 (0.21–0.70)	0.005	0.003	0.083
Self-reported CVD	0.65 (0.37–1.15)	0.43 (0.17–1.10)	0.163	0.069	0.52 (0.27–0.99)	0.70 (0.37–1.33)	0.134	0.285	0.207

CI, confidence interval, CVD, cardiovascular disease; IPAQ, Short-form International Physical Activity Questionnaire; MVPA, moderate-to-vigorous physical activity; PASBAQ, Physical Activity and Sedentary Behaviour Assessment Questionnaire; WEMWBS Warwick-Edinburgh Mental Well-Being Scale.

Estimates age-standardised using the 2012 English household population.

^a^ Odds ratios obtained using logistic regression, with the health outcome as dependent variable and tertiles of MVPA as a categorical variable (lowest group as the reference).

^b^ *P*-value for trend obtained using logistic regression, with the health outcome as dependent variable and tertiles of MVPA entered as a single continuous independent variable.

^c^ PASBAQ- and IPAQ-MVPA included in the same model (adjusted for age) as continuous independent variables; *P*-value shown is the test for statistical interaction.

**Fig 6:**
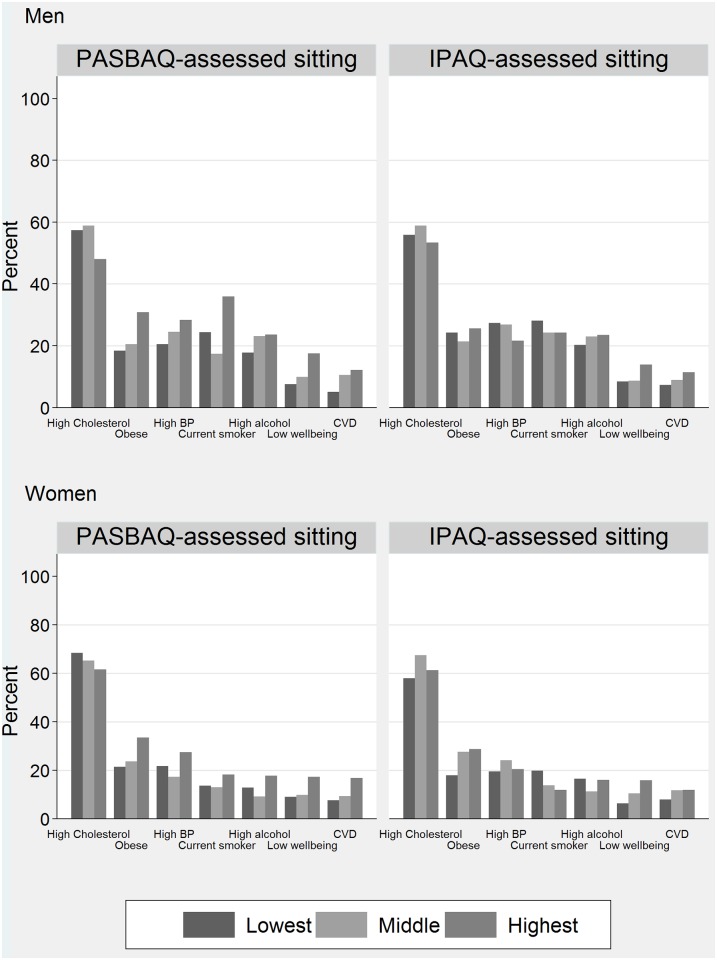
Prevalence of health outcomes according to the PASBAQ- and Short-form IPAQ-assessed tertiles of time spent sitting for men (top panel) and for women (lower panel). Proportion of participants categorised with physical health and mental health outcomes according to the Physical Activity and Sedentary Behaviour Assessment Questionnaire (PASBAQ) and Short-form International Physical Activity Questionnaire (IPAQ) assessed tertiles of time spent sitting on weekdays.

**Fig 7:**
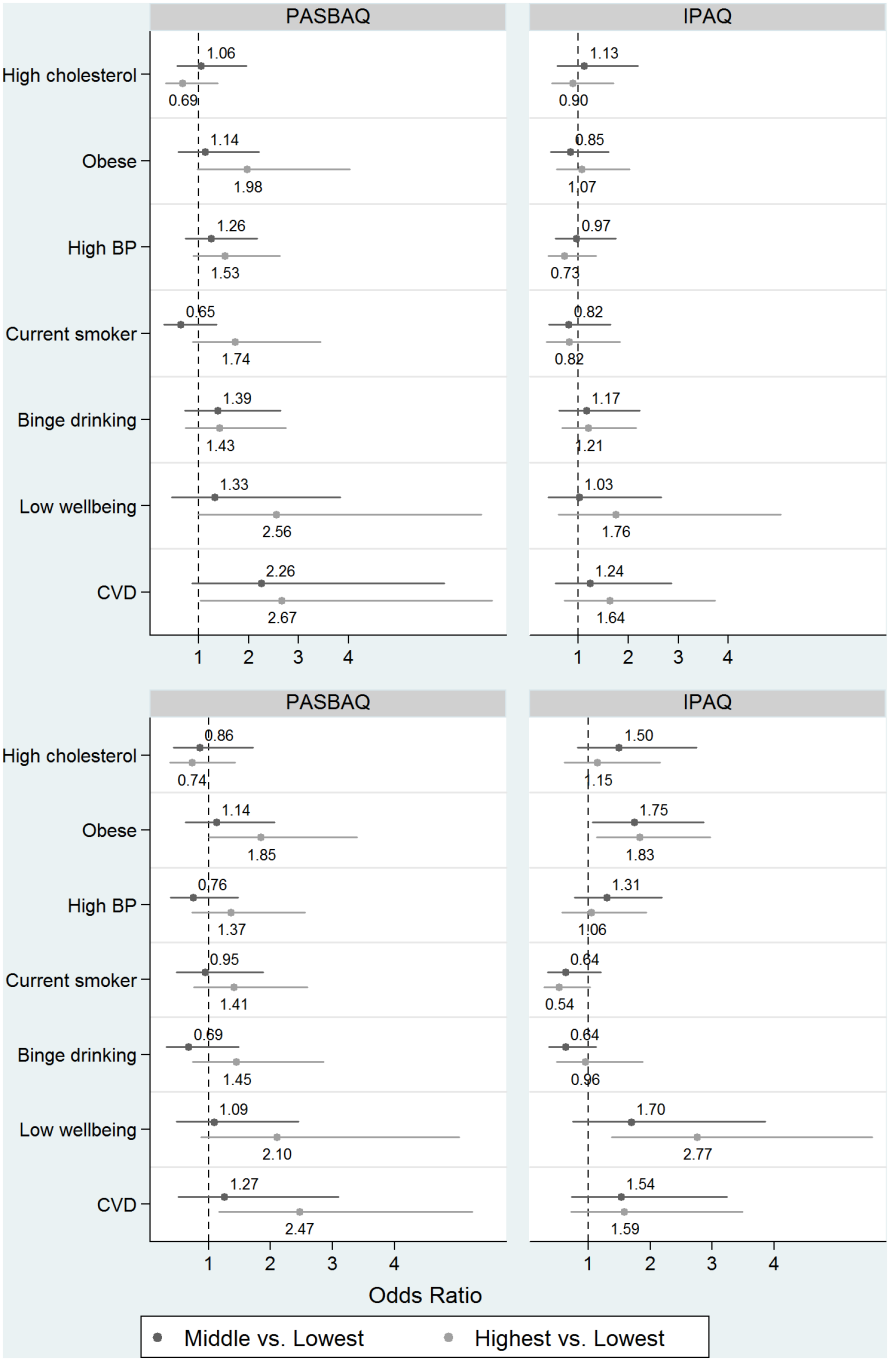
Associations of PASBAQ- and IPAQ-assessed time spent sitting with odds of unfavourable health outcomes for men (top panel) and women (lower panel). The odds ratios (and 95% CI) shown in Table 7 are shown in graphical form. The estimates compare participants in: (1) the middle tertile of sitting time *vs*. lowest tertile of sitting time (dark grey), and (2) the highest tertile of sitting time *vs*. lowest tertile of sitting time (light grey), grouped by health outcome.

Fig 6 shows the age-standardised prevalence estimates of health outcomes according to the PASBAQ- and IPAQ-assessed tertiles of time spent sitting. Table 7 shows the corresponding odds ratios with the *P*-values for group differences and for linear trend. Using PASBAQ data, more time spent sitting on weekdays was associated with increased odds of: being classed as obese (men: *P* for trend = 0.063; women: *P* for trend = 0.044), having a low WEMWBS score (men: *P* for trend = 0.053), and reporting CVD (men: *P* for trend = 0.032; women: *P* for trend = 0.012). IPAQ-assessed sitting showed graded associations with health outcomes for women but not for men. For women, more time spent sitting was associated with increased odds of being classed as obese (*P* for trend = 0.014) and increased odds of having a low WEMWBS score (*P* for trend = 0.004) (Fig 7). Null associations across both questionnaires were found for excessive alcohol consumption, hypertension, and raised cholesterol. P-values for the PASBAQ × IPAQ interaction term did not reach statistical significance for any health outcome.

**Table 7:** Associations of PASBAQ- and IPAQ-assessed time spent sitting on weekdays with odds of unfavourable health outcomes.

Health outcomes	PASBAQ-sitting time				IPAQ-sitting time				PASBAQ and IPAQ
	Middle	Highest	*P*^a^	*P*^b^	Middle	Highest	*P*^a^	*P*^b^	*P*^c^
	Odds ratio (95% CI)				Odds ratio (95% CI)				
**Men**									
Raised cholesterol	1.06 (0.58–1.96)	0.69 (0.34–1.39)	0.419	0.324	1.13 (0.58–2.19)	0.90 (0.48–1.71)	0.769	0.689	0.713
Obese	1.14 (0.59–2.20)	1.98 (0.97–4.02)	0.136	0.063	0.85 (0.45–1.61)	1.07 (0.57–2.03)	0.716	0.767	0.366
Hypertension	1.26 (0.73–2.17)	1.53 (0.89–2.63)	0.299	0.119	0.97 (0.54–1.76)	0.73 (0.40–1.36)	0.476	0.294	0.296
Current smoker	0.65 (0.31–1.36)	1.74 (0.88–3.44)	0.007	0.135	0.82 (0.41–1.65)	0.82 (0.37–1.83)	0.847	0.662	0.066
Above alcohol limits	1.39 (0.73–2.63)	1.43 (0.74–2.75)	0.466	0.286	1.17 (0.62–2.22)	1.21 (0.68–2.15)	0.801	0.530	0.438
Low WEMWBS	1.33 (0.46–3.83)	2.56 (0.99–6.66)	0.127	0.053	1.03 (0.40–2.66)	1.76 (0.61–5.05)	0.396	0.256	0.632
Self-reported CVD	2.26 (0.87–5.91)	2.67 (1.04–6.87)	0.124	0.032	1.24 (0.54–2.86)	1.64 (0.72–3.74)	0.450	0.214	0.134
**Women**									
Raised cholesterol	0.86 (0.43–1.72)	0.74 (0.38–1.43)	0.634	0.363	1.50 (0.83–2.74)	1.15 (0.62–2.16)	0.380	0.689	0.074
Obese	1.14 (0.63–2.06)	1.85 (1.01–3.40)	0.074	0.044	1.75 (1.07–2.86)	1.83 (1.13–2.97)	0.034	0.014	0.943
Hypertension	0.76 (0.39–1.48)	1.37 (0.73–2.56)	0.133	0.293	1.31 (0.78–2.19)	1.06 (0.58–1.94)	0.494	0.892	0.277
Current smoker	0.95 (0.48–1.88)	1.41 (0.77–2.60)	0.429	0.265	0.64 (0.34–1.20)	0.54 (0.28–1.03)	0.169	0.070	0.594
Above alcohol limits	0.69 (0.32–1.49)	1.45 (0.74–2.86)	0.082	0.257	0.64 (0.36–1.13)	0.96 (0.49–1.88)	0.257	0.960	0.132
Low WEMWBS	1.09 (0.49–2.45)	2.10 (0.88–5.04)	0.150	0.093	1.70 (0.75–3.85)	2.77 (1.37–5.57)	0.015	0.004	0.651
Self-reported CVD	1.27 (0.52–3.10)	2.47 (1.16–5.26)	0.014	0.012	1.54 (0.73–3.24)	1.59 (0.72–3.49)	0.470	0.241	0.815

CI, confidence interval, CVD, cardiovascular disease; IPAQ, Short-form International Physical Activity Questionnaire; MVPA, moderate-to-vigorous physical activity; PASBAQ, Physical Activity and Sedentary Behaviour Assessment Questionnaire; WEMWBS Warwick-Edinburgh Mental Well-Being Scale.

Estimates age-standardised using the 2012 English household population.

^a^ Odds ratios obtained using logistic regression, with the health outcome as dependent variable and tertiles of MVPA as a categorical variable (lowest group as the reference).

^b^ *P*-value for trend obtained using logistic regression, with the health outcome as dependent variable and tertiles of MVPA entered as a single continuous independent variable.

^c^ PASBAQ- and IPAQ-MVPA included in the same model (adjusted for age) as continuous independent variables; *P*-value shown is the test for statistical interaction.

### Sensitivity analyses

S1 Table presents PASBAQ-assessed average MPVA and average sitting time for the PASBAQ without IPAQ and PASBAQ with IPAQ groups within each propensity score quintile. Differences in average MVPA and average sitting time were statistically independent of the estimated propensity to have completed both instruments. On average, the difference in MVPA between the two groups (PASBAQ with IPAQ—PASBAQ without IPAQ) was 7.5 minutes/week (95% CI: -133.0 to 147.9) in the first quintile (highest propensity) and -25.0 minutes/week (95% CI: -100.5 to 50.4) in the fifth quintile. Equivalent figures for time spent sitting were 14.1 minutes/weekday (95% CI: -16.3 to 44.5) and -1.2 minutes/weekday (95% CI: -25.2 to 22.8).

Excluding all walking from MVPA narrowed the gap in prevalence estimates, but the IPAQ-based estimates of sufficient aerobic activity remained higher than the PASBAQ (S1 Fig). Prevalence estimates of inactivity also remained lower using the IPAQ. Kappa and PABAK statistics were similar in magnitude for sufficient aerobic activity and for inactivity, with the strength of agreement being fair-to-moderate (PABAK: 0.32 to 0.44) and moderate (PABAK: 0.42 to 0.57) respectively (S2 Table). The dose-response associations between MVPA and other health outcomes were not sensitive to the treatment of walking for men (S3 Table). Among women, associations between MVPA and health were not sensitive to the treatment of walking using the IPAQ. Using PASBAQ data, excluding walking from MVPA attenuated the dose-response association with obesity but strengthened the association with current smoking and drinking above recommended daily alcohol limits on the heaviest drinking day in the last 7 days. Excluding occupational physical activity from MVPA showed little change in the strength of relative agreement (S2 Fig; S4 and S5 Tables).

## Discussion

Validation studies have compared self-reported data on physical activity and sedentary behaviour with device-based methods such as accelerometry [22,56] and physical activity related energy-expenditure through the doubly-labelled water method [31]. The majority of studies have shown positive but moderate associations between reported and device-based methods [58]. Questionnaires remain the most feasible method to assess levels of physical activity and sedentary behaviour at the population level due in part to the expensive costs and high respondent burden associated with using device-based methods within large-scale health examination surveys. Reported methods are also the measurement tool on which current UK health-based recommendations have been made. While reported methods are more feasible than device-based methods, questionnaire space in large-scale surveys is expensive and limited, leading to continued efforts to develop shorter instruments that produce comparable data to longer, more detailed instruments. For this application, the most important correlation is that between questionnaires, not their level of agreement with device-based methods. In this study we compared data obtained from a long- and short-physical activity questionnaire (PASBAQ and IPAQ respectively) administered to the same sample to examine the usefulness of including the shorter instrument in future annual rounds of the HSE to complement occasional use of the longer instrument.

IPAQ-assessed prevalence estimates of sufficient aerobic activity (MVPA ≥150minutes/week) and inactivity (MVPA<30minutes/week) were higher and lower respectively than the PASBAQ. IPAQ-assessed estimates of excessive sitting (sitting ≥540minutes/weekday) were also higher. Demographic patterns in prevalence estimates were similar. PABAK statistics showed fair-to-moderate agreement for sufficient aerobic activity (ranging from 0.32 to 0.49), moderate-to-substantial agreement for inactivity (0.42 to 0.74), and moderate-to-substantial agreement for excessive sitting (0.49 to 0.75). Agreement based on the PABAK varied across population subgroups for activity and for inactivity, being highest among groups with the highest volumes of MVPA. As with PASBAQ data, IPAQ-assessed MVPA showed graded associations with having a low score on a positive mental well-being scale (WEMWBS), obesity, and reported CVD. IPAQ-assessed sitting showed graded associations with positive mental well-being and obesity for women but not for men. Higher estimates of adherence to physical activity recommendations using the Short-form IPAQ compared with longer, more detailed instruments was also found in similar studies conducted in the United States [33] and Australia [59].

### Explanations for difference in prevalence

Different physical activity questionnaires administered to the same sample produce varying prevalence estimates because of differences in questionnaire structure and content rather than actual differences in reported physical activity and sedentary behaviour [33,60]. Given the complexity of these behaviours, and their several dimensions, data from shorter, brief questionnaires such as the Short-form IPAQ will not correlate exactly with data from longer instruments, in part because all activity domains (e.g., occupational and leisure-time) are reported in aggregate, and that short, all-encompassing questions are likely to be the most cognitively challenging for participants to accurately comprehend and formulate a reasonable response to [32,61]. Our study highlighted a number of differences between the two instruments that go some way to explaining the gap in prevalence estimates, and the slight-to-moderate (aerobic activity) and moderate-to-substantial (inactivity and excessive sitting) levels of relative agreement. First, the PASBAQ and Short-form IPAQ differ in the duration of recall for physical activity (28 *vs*. 7 days respectively). This is exacerbated when the index date of completing the questionnaire differs, as occurred in this study. Secondly, the exclusion / inclusion of work-based activities in the PASBAQ and IPAQ respectively partly explains the higher IPAQ-assessed estimates of sufficient aerobic activity and excessive sitting, and lower estimates of inactivity. Thirdly, the different approaches to capturing intensity (PASBAQ: MET compendium [38,39] and follow-up questions on breathing; IPAQ: participants self-report activities as either vigorous or moderate with the aid of examples and physiological cues) may also partly explain the gap in the prevalence estimates based on cut-points for the weekly volume of MVPA. The reliance of the IPAQ on participants to make their own judgements about the intensity of their activities has been argued to lead to potential “spill-over effects”, where participants report relatively light-intensity activities as moderate-intensity, and report moderate-intensity activities as vigorous. The placement of vigorous- before moderate-intensity items in the IPAQ has also been identified as a possible source of double-counting of activity [32]. These features of the IPAQ are particularly relevant to the assessment of adherence to current MVPA-based recommendations for aerobic activity which give vigorous-intensity activities twice the credit of moderate-intensity activities [15]. Finally, the differential treatment of walking (PASBAQ: exclusion of slow or average-paced walking from MVPA; IPAQ: intensity of walking not assessed, and so in our primary analysis we assumed all walking to be of at least moderate-intensity) also partly explains the higher estimates of sufficient aerobic activity, and lower estimates of inactivity, obtained using IPAQ data. Analysis of PASBAQ data showed that 56% of participants who reported having done a continuous walk lasting for over five minutes in the last 28 days reported their walking-pace to be slow or average (data not shown). The gap in prevalence estimates for activity and for inactivity shown in this study means that the Kappa values for 2×2 tables should be interpreted with caution [51].

### Modifications to the shorter questionnaire

Following our comparison study, four main modifications were made to the current version of the Short-form IPAQ (for inclusion in HSE 2015). First, the order of questions was reversed so that questions on walking appeared first, followed by moderate- and then vigorous-activities in order to minimise both spill-over effects and the potential double-counting of activities. Secondly, two questions about walking-pace and effort (taken from the PASBAQ) were added to collect data on intensity. Including them will enable data-users to distinguish between light- and moderate-intensity walking in the same way as with PASBAQ data (i.e., walking is counted as a moderate-intense activity if participants of any age report walking at a ‘fairly brisk’ or ‘fast’ usual pace, and for those aged ≥65years for whom ‘average’ or ‘slow’ paced walking made them *“breathe faster*, *feel warmer*, *or sweat”*). We would anticipate that excluding light-intensity walking from MVPA would result in lower prevalence estimates of sufficient aerobic activity, and higher estimates of inactivity, than those shown in this study; it would also improve the classification of participants across broad categories of MVPA. A recent analysis of UK Biobank data showed self-reported walking-pace to be a strong predictor of all-cause mortality [62], and secondary analysis of HSE data showed walking at a brisk or fast pace to be the strongest aspect of physical activity associated with various measures of weight [63], illustrating the value of adding the question on walking pace to the annual HSE. Thirdly, the examples of moderate- and vigorous-activities were updated to more closely align with the examples in the PASBAQ. Finally, the word ‘average’ was added to the single-item on weekday sitting to minimise the possibility that participants mistakenly report a weekly total (*“During the last 7 days*, *how much time did you spend sitting on an average weekday*?*”*). An example was also added to illustrate how participants should report their answer.

### Strengths and limitations

A main strength of our study was the large sample, allowing comparisons across subgroups based on gender, age, socioeconomic status, and objective measures of body mass index, blood pressure, and resting heart rate. Participants completed both questionnaires, thus affording direct comparisons. Definitions of sufficient aerobic activity, inactivity, and excessive sitting were consistent across both instruments, eliminating differences in cut-points as an explanation for differences in prevalence estimates. Time spent in MVPA and time spent sitting on weekdays were measured on a continuous scale, allowing the use of percentile groups to better examine dose-response associations with a range of physical health and mental health variables.

Our study had a number of limitations. In the HSE 2012, the PASBAQ and IPAQ were positioned in the main interview and nurse-visit respectively, and so were administered on average one month apart. Participant responses to the IPAQ may have been influenced by their earlier responses to the PASBAQ, e.g. participants may have modified their behaviour in the period of time between instruments, or have been influenced by the context of other questions. A crossover design—in which participants are randomised to a balanced ordering of the instruments (long-form followed by short-form; short-form followed by long-form)–would have enabled us to control for an order effect when comparing the instruments. However, a crossover design was not possible in our study as it is essential to use a standardised protocol to administer the PASBAQ for the purposes of using HSE data to monitor changes over time in adherence to UK physical activity recommendations. We examined the sensitivity of our results by conducting a multiple linear regression analysis of the difference in time spent in MVPA (PASBAQ—IPAQ) using the number of days between data collection points as an independent variable adjusted for age and sex. The number of days between data collection points was not a significant predictor of the difference in MVPA (*P* = 0.972, data not shown), suggesting that behaviour change between the PASBAQ and IPAQ did not materially influence our findings. As the IPAQ was administered only in the fourth quarter of fieldwork, spanning the winter months, the findings of our study cannot be assumed to be generalizable to the full HSE year. Although the analytical sample was older on average than the rest of the HSE sample, it did not differ with regard to other socio-demographic characteristics such as gender, socioeconomic status, and self-reported health conditions. The older age of the analytical sample slightly reduced the representativeness of our data, but it did not influence our findings as we compared the two instruments using data collected from the same sample. Finally, the cross-sectional nature of the study precludes us from making any inferences about direction or causality.

The existing Short-form IPAQ was included in HSE 2013–14. The modified Short-form IPAQ was included in HSE 2015. The Health and Social Care Information Centre is currently considering the inclusion of the modified Short-form IPAQ in the core content of future annual rounds of HSE (from 2016 onwards). This complements the detailed information collected by the PASBAQ at approximately 5-yearly intervals. This enables descriptive analysis of broad discrete categories of physical activity and sedentary behaviour, and its cross-sectional associations with health. For example, its inclusion in HSE 2013 enabled analysis of the association between shift-working and physical activity (grouped into tertiles according to the total amount of weekly activity reported) [64].

## Conclusions

Feasibility and costs are important considerations for choosing self-report or device-based methods to assess physical activity or sedentary behaviour. Despite decreasing costs for device-based measures, reported methods remain less expensive than device-based methods, especially for large studies. Obtaining high quality data from reported methods requires choosing the right instruments and using them correctly. Capturing the multi-dimensional nature of habitual physical activity and sedentary behaviour through brief questionnaires is complex. Differences in prevalence estimates can reflect differences in questionnaire structure and content—and the analytical assumptions they impose on the data—rather than differences in reported behaviour. Treating all IPAQ-assessed walking as moderate-intensity contributed to the differences in prevalence estimates based on thresholds of MVPA, and the fair-to-moderate strength of agreement. PASBAQ data will continue to be used for population surveillance at 4- to 5-yearly intervals. The Short-form IPAQ was included in HSE 2013–14 to enable more frequent assessment of physical activity and sedentary behaviour; a modified version with different item-ordering and additional questions on walking-pace and effort was included in HSE 2015.

## Supporting Information

S1 Fig Prevalence of health outcomes according to the PASBAQ- and Short-form IPAQ-assessed tertiles of MVPA excluding all walking. This figure compares the proportion of participants categorised with physical health and mental health outcomes according to the PASBAQ and Short-form IPAQ assessed tertiles of time MVPA excluding all walking, by gender, age-group, equivalised household income, resting pulse rate (RHR), and BMI status.
(TIF)

S2 Fig Prevalence of health outcomes according to the PASBAQ- and Short-form IPAQ-assessed tertiles of MVPA excluding occupational physical activity. This figure compares the proportion of participants categorised with physical health and mental health outcomes according to the PASBAQ and Short-form IPAQ assessed tertiles of time spent in MVPA excluding occupational physical activity, by gender, age-group, income, resting pulse rate (RHR), and BMI category.
(TIF)

S1 Table PASBAQ-assessed MVPA and Sitting time for participants in the PASBAQ without IPAQ and PASBAQ with IPAQ groups, by quintile of propensity score. (DOCX)

S2 Table Kappa statistic and 95% CI, and the prevalence-adjusted bias-adjusted Kappa (PABAK) statistics for PASBAQ- and IPAQ-based estimates of sufficient aerobic activity and inactivity excluding time spent walking from MVPA. (DOCX)

S3 Table Associations of PASBAQ- and IPAQ-assessed time spent in MVPA with odds of unfavourable health outcomes. Estimates of MVPA excluded walking.
(DOCX)

S4 Table Kappa statistic and 95% CI, and the prevalence-adjusted bias-adjusted Kappa (PABAK) statistics for PASBAQ- and IPAQ-based estimates of sufficient aerobic activity and inactivity excluding occupational activity from MVPA. (DOCX)

S5 Table Associations of PASBAQ- and IPAQ-assessed time spent in MVPA with odds of unfavourable health outcomes. Estimates of MVPA excluded occupational physical activity.
(DOCX)
