# Supplementary figures and images for: Comparison of the Physical Activity and Sedentary Behaviour Assessment Questionnaire and the Short-Form International Physical Activity Questionnaire: An Analysis of Health Survey for England Data
Source: PLoS One. 2016 Mar 18;11(3):e0151647. doi: 10.1371/journal.pone.0151647 (PMC4798726; doi:10.1371/journal.pone.0151647)

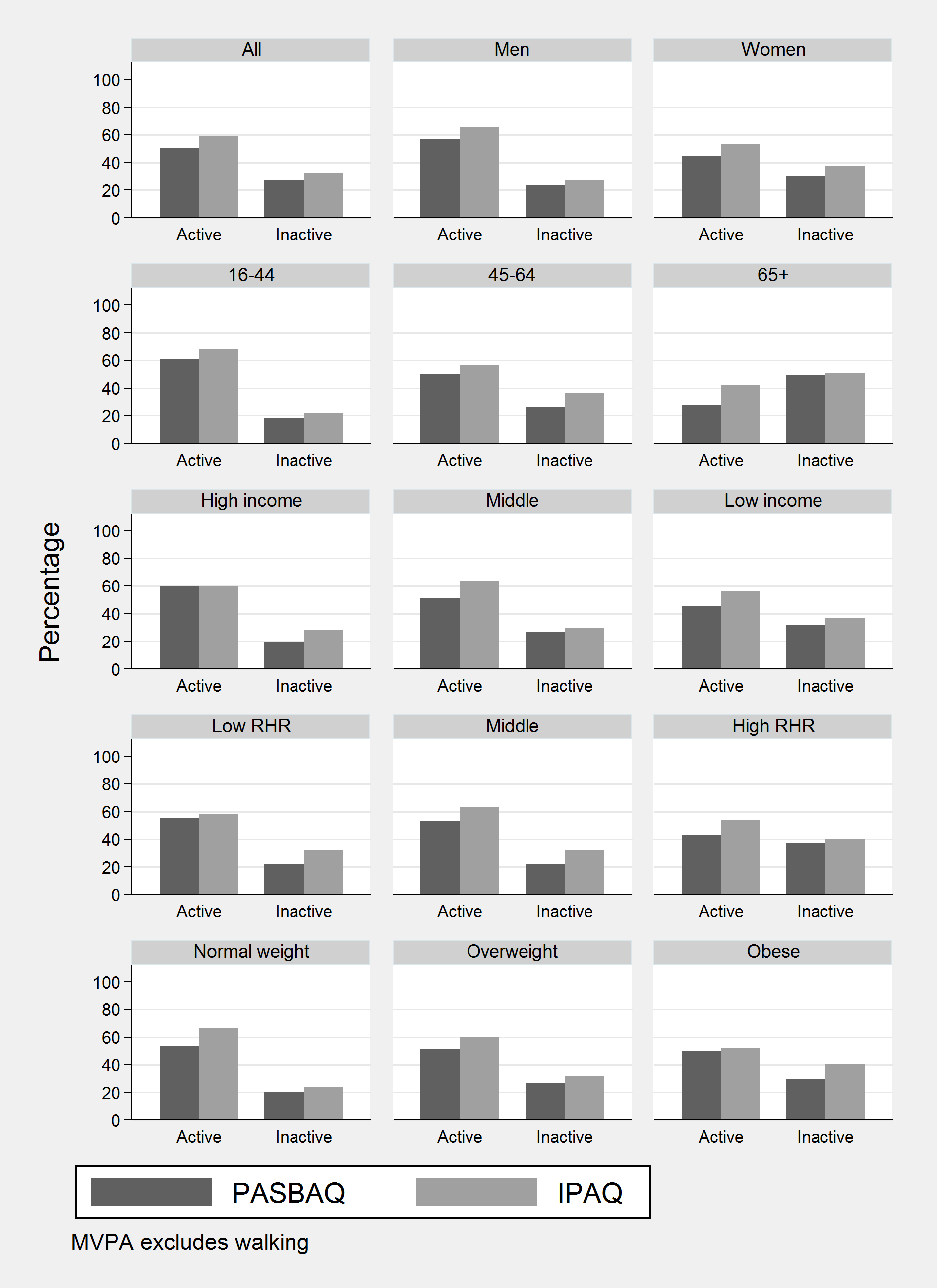

Supplement: S1 Fig — This figure compares the proportion of participants categorised with physical health and mental health outcomes according to the PASBAQ and Short-form IPAQ assessed tertiles of time MVPA excluding all walking, by gender, age-group, equivalised household income, resting pulse rate (RHR), and BMI status. (TIF) [file pone.0151647.s001.tif]

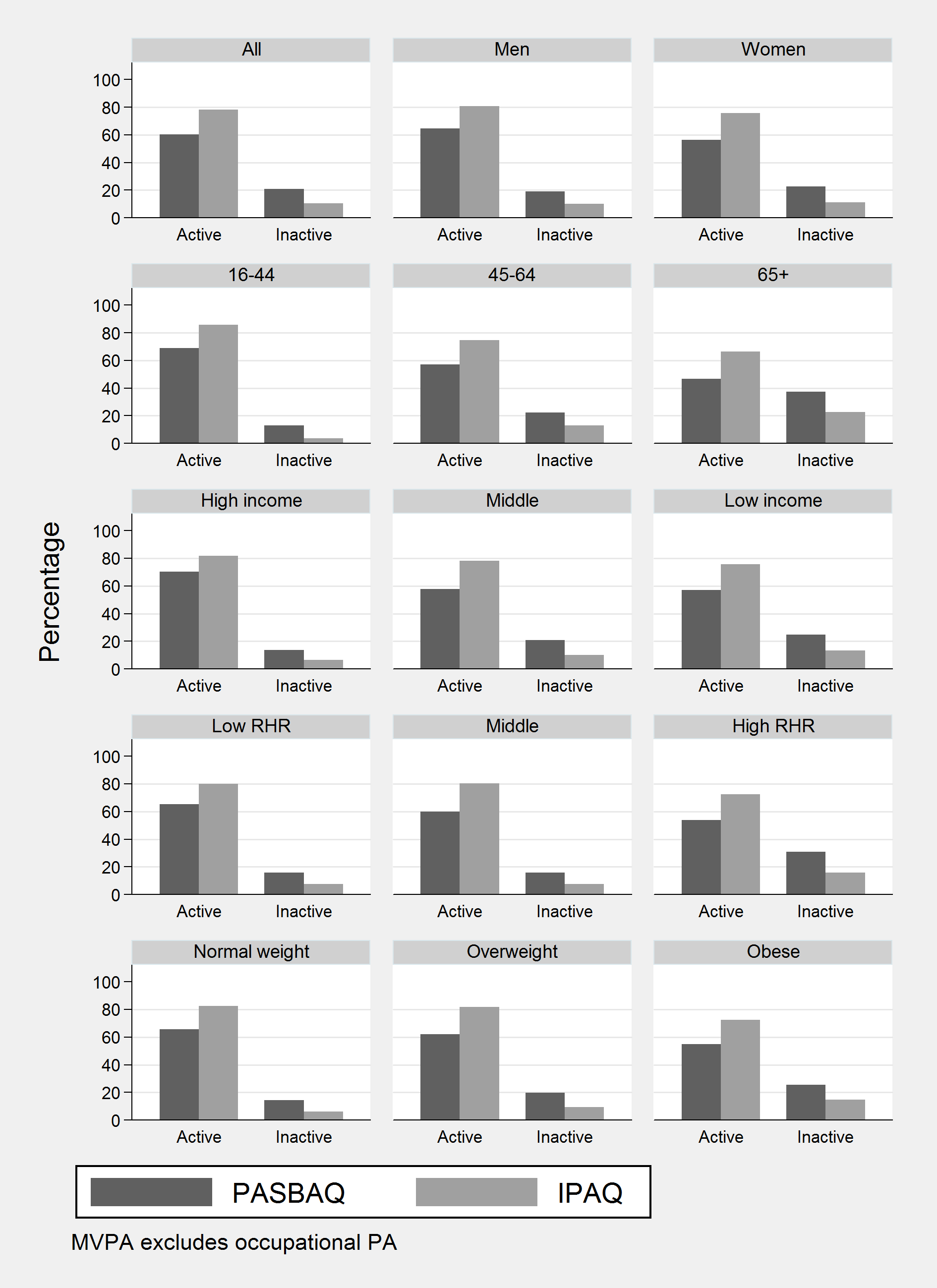

Supplement: S2 Fig — This figure compares the proportion of participants categorised with physical health and mental health outcomes according to the PASBAQ and Short-form IPAQ assessed tertiles of time spent in MVPA excluding occupational physical activity, by gender, age-group, income, resting pulse rate (RHR), and BMI category. (TIF) [file pone.0151647.s002.tif]
