# Supplementary material for: Comparison of the Physical Activity and Sedentary Behaviour Assessment Questionnaire and the Short-Form International Physical Activity Questionnaire: An Analysis of Health Survey for England Data
Source: PLoS One. 2016 Mar 18;11(3):e0151647. doi: 10.1371/journal.pone.0151647 (PMC4798726; doi:10.1371/journal.pone.0151647)
Supplement: S1 Table — (DOCX) [file pone.0151647.s003.docx]

**S1 Table PASBAQ-assessed MVPA and Sitting time for participants in the PASBAQ without IPAQ and PASBAQ with IPAQ groups, by quintile of propensity score**

| **Response propensity** | **MVPA (minutes/week)** | | | | **Sitting (minutes/weekday)** | | | |
| --- | --- | --- | --- | --- | --- | --- | --- | --- |
|  | **PASBAQ without IPAQ** (*n*=6921) | **PASBAQ with IPAQ** (*n*=1252) | **Difference (95% CI)** | **P-value** | **PASBAQ without IPAQ** (*n*=6921) | **PASBAQ with IPAQ** (*n*=1252) | **Difference (95% CI)** | **P-value** |
| **Quintile 1 (highest)** | 560.4 | 567.9 | 7.5  (-133.0, 147.9) | 0.917 | 289.0 | 303.0 | 14.1  (-16.3, 44.5) | 0.364 |
| **Quintile 2** | 529.6 | 567.1 | 37.5  (-65.7, 140.7) | 0.476 | 279.7 | 297.5 | 17.8  (-7.8, 43.5) | 0.173 |
| **Quintile 3** | 534.1 | 497.2 | -36.9  (-126.4, 52.6) | 0.419 | 266.9 | 285.6 | 18.7  (-2.0, 39.5) | 0.077 |
| **Quintile 4** | 522.4 | 550.6 | 28.3  (-70.9, 127.4) | 0.576 | 289.8 | 288.7 | -1.2  (-22.1, 19.8) | 0.913 |
| **Quintile 5 (lowest)** | 424.6 | 399.6 | -25.0  (-100.5, 50.4) | 0.515 | 314.1 | 312.9 | -1.2  (-25.2, 22.8) | 0.919 |

CI, confidence interval, IPAQ, Short-form International Physical Activity Questionnaire; MVPA, moderate-to-vigorous physical activity; PASBAQ, Physical Activity and Sedentary Behaviour Assessment Questionnaire.

Logistic regression used to estimate the propensity score: the dependent variable was sample type (0 = PASBAQ without IPAQ; 1 = PASBAQ with IPAQ), with sex, age, region, number of adults and children in the household, BMI, marital status, income tertiles, presence of CVD, smoking status, adherence to NHS recommended daily alcohol limits, National Statistics Socio-Economic Classification, and the main interview non-response weight as independent variables. Multiple imputation was used to replace missing values. We carried out 10 imputations. Parameter estimates were combined across the imputed datasets by using Rubin’s rules.
