# Supplementary material for: Comparison of the Physical Activity and Sedentary Behaviour Assessment Questionnaire and the Short-Form International Physical Activity Questionnaire: An Analysis of Health Survey for England Data
Source: PLoS One. 2016 Mar 18;11(3):e0151647. doi: 10.1371/journal.pone.0151647 (PMC4798726; doi:10.1371/journal.pone.0151647)
Supplement: S2 Table — (DOCX) [file pone.0151647.s004.docx]

**S2 Table** Kappa statistic and 95% CI, and the prevalence-adjusted bias-adjusted Kappa (PABAK) statistic for PASBAQ- and IPAQ-based estimates of sufficient aerobic activity and inactivity excluding time spent walking from MVPA

|  | **Sufficient activity**  **(MVPA ≥150minutes/weekday)** | | | | | **Inactivity**  **(MVPA <30minutes/weekday)** | | | | |
| --- | --- | --- | --- | --- | --- | --- | --- | --- | --- | --- |
|  | **Kappa**  **(95% CI)** | **K_max_** | **PABAK** | **PI** | **BI** | **Kappa**  **(95% CI)** | **K_max_** | **PABAK** | **PI** | **BI** |
| **All** | 0.38 (0.33-0.44) | 0.83 | 0.38 | -0.10 | 0.09 | 0.40 (0.34-0.45) | 0.87 | 0.49 | 0.41 | 0.05 |
| **Sex:** |  |  |  |  |  |  |  |  |  |  |
| Men | 0.41 (0.33-0.50) | 0.82 | 0.44 | -0.22 | 0.08 | 0.38 (0.29-0.47) | 0.91 | 0.53 | 0.49 | 0.03 |
| Women | 0.33 (0.26-0.41) | 0.83 | 0.33 | 0.02 | 0.09 | 0.40 (0.33-0.47) | 0.84 | 0.46 | 0.33 | 0.07 |
| **Age-group:** |  |  |  |  |  |  |  |  |  |  |
| 16-44 | 0.38 (0.29-0.48) | 0.83 | 0.43 | -0.29 | 0.08 | 0.31 (0.20-0.42) | 0.88 | 0.56 | 0.60 | 0.04 |
| 45-64 | 0.32 (0.23-0.41) | 0.87 | 0.32 | -0.06 | 0.07 | 0.36 (0.26-0.45) | 0.77 | 0.44 | 0.37 | 0.10 |
| 65+ | 0.33 (0.23-0.43) | 0.69 | 0.37 | 0.31 | 0.14 | 0.44 (0.34-0.53) | 0.98 | 0.44 | 0.00 | 0.01 |
| **BMI group:** |  |  |  |  |  |  |  |  |  |  |
| Normal | 0.37 (0.27-0.47) | 0.74 | 0.38 | -0.21 | 0.13 | 0.36 (0.24-0.48) | 0.91 | 0.56 | 0.55 | 0.03 |
| Overweight | 0.36 (0.27-0.45) | 0.84 | 0.36 | -0.11 | 0.08 | 0.30 (0.20-0.40) | 0.88 | 0.42 | 0.42 | 0.05 |
| Obese | 0.36 (0.25-0.48) | 0.96 | 0.37 | -0.02 | 0.02 | 0.44 (0.33-0.55) | 0.77 | 0.49 | 0.30 | 0.11 |
| **Income:** |  |  |  |  |  |  |  |  |  |  |
| Highest | 0.37 (0.27-0.47) | 0.77 | 0.35 | -0.02 | 0.11 | 0.42 (0.31-0.53) | 0.90 | 0.51 | 0.31 | 0.05 |
| Middle | 0.38 (0.29-0.48) | 0.74 | 0.39 | -0.15 | 0.13 | 0.34 (0.22-0.45) | 0.93 | 0.46 | 0.44 | 0.03 |
| Lowest | 0.36 (0.25-0.48) | 1.00 | 0.40 | -0.20 | 0.00 | 0.46 (0.35-0.57) | 0.76 | 0.57 | 0.52 | 0.09 |
| **Heart rate:** |  |  |  |  |  |  |  |  |  |  |
| Lowest | 0.38 (0.27-0.48) | 0.94 | 0.39 | -0.14 | 0.03 | 0.39 (0.28-0.51) | 0.76 | 0.52 | 0.46 | 0.10 |
| Middle | 0.36 (0.26-0.47) | 0.79 | 0.38 | -0.16 | 0.10 | 0.39 (0.28-0.50) | 0.92 | 0.54 | 0.50 | 0.03 |
| Highest | 0.41 (0.31-0.51) | 0.78 | 0.40 | 0.03 | 0.11 | 0.42 (0.32-0.52) | 0.93 | 0.44 | 0.23 | 0.01 |

BI, bias-index; BMI, body mass index; CI, confidence interval; IPAQ, Short-form International Physical Activity Questionnaire; κ_max_, maximum attainable value of the Kappa statistic; MVPA, moderate-to-vigorous physical activity; PABAK, prevalence-adjusted bias-adjusted Kappa statistic; PASBAQ, physical activity and sedentary behaviour questionnaire; PI, prevalence-index.

Bias-index denotes the difference between disagreements; Prevalence-index denotes the difference between agreements on the positive and negative classification.
