# Supplementary material for: Comparison of the Physical Activity and Sedentary Behaviour Assessment Questionnaire and the Short-Form International Physical Activity Questionnaire: An Analysis of Health Survey for England Data
Source: PLoS One. 2016 Mar 18;11(3):e0151647. doi: 10.1371/journal.pone.0151647 (PMC4798726; doi:10.1371/journal.pone.0151647)
Supplement: S3 Table — Estimates of MVPA excluded walking. (DOCX) [file pone.0151647.s005.docx]

**S3 Table** Associations of PASBAQ- and IPAQ-assessed time spent in MVPA with odds of unfavourable health outcomes. Estimates of MVPA excluded walking.

| **Health outcomes** | **PASBAQ-MVPA** | | | |  | **IPAQ-MVPA** | | | |  | **PASBAQ and IPAQ** |
| --- | --- | --- | --- | --- | --- | --- | --- | --- | --- | --- | --- |
|  | **Middle** | **Highest** | *P*^a^ | *P*^b^ |  | **Middle** | **Highest** | *P*^a^ | *P*^b^ |  | *P*^c^ |
|  | **Odds ratio (95% CI)** | |  |  |  | **Odds ratio (95% CI)** | |  |  |  |  |
| **Men** |  |  |  |  |  |  |  |  |  |  |  |
| Raised cholesterol | 0.37 (0.19-0.73) | 0.52 (0.25-1.09) | 0.019 | 0.158 |  | 0.55 (0.30-1.00) | 0.80 (0.45-1.43) | 0.136 | 0.583 |  | 0.866 |
| Obese | 0.89 (0.45-1.79) | 0.98 (0.51-1.88) | 0.943 | 0.963 |  | 0.58 (0.32-1.05) | 0.86 (0.47-1.55) | 0.177 | 0.727 |  | 0.901 |
| Hypertension | 0.86 (0.49-1.49) | 0.84 (0.46-1.51) | 0.800 | 0.564 |  | 0.63 (0.35-1.15) | 0.83 (0.44-1.57) | 0.295 | 0.631 |  | 0.284 |
| Current smoker | 1.00 (0.55-1.84) | 0.84 (0.47-1.47) | 0.759 | 0.511 |  | 0.69 (0.36-1.29) | 0.87 (0.47-1.62) | 0.469 | 0.729 |  | 0.866 |
| Above alcohol limits | 1.13 (0.50-2.59) | 0.92 (0.42-2.00) | 0.735 | 0.797 |  | 0.90 (0.44-1.85) | 0.99 (0.50-1.93) | 0.938 | 0.992 |  | 0.911 |
| Low WEMWBS | 0.35 (0.16-0.77) | 0.24 (0.09-0.62) | 0.002 | 0.003 |  | 0.36 (0.15-0.88) | 0.52 (0.21-1.24) | 0.069 | 0.184 |  | 0.186 |
| Self-reported CVD | 0.92 (0.50-1.69) | 0.62 (0.27-1.43) | 0.529 | 0.245 |  | 0.76 (0.40-1.43) | 0.49 (0.25-0.96) | 0.116 | 0.036 |  | 0.940 |
| **Women** |  |  |  |  |  |  |  |  |  |  |  |
| Raised cholesterol | 1.18 (0.66-2.10) | 0.57 (0.29-1.11) | 0.016 | 0.090 |  | 0.97 (0.57-1.65) | 0.89 (0.47-1.68) | 0.924 | 0.710 |  | 0.341 |
| Obese | 0.91 (0.57-1.47) | 0.75 (0.43-1.33) | 0.605 | 0.317 |  | 0.52 (0.32-0.85) | 0.49 (0.30-0.79) | 0.007 | 0.004 |  | 0.223 |
| Hypertension | 1.16 (0.70-1.90) | 1.38 (0.57-3.33) | 0.689 | 0.497 |  | 0.68 (0.38-1.24) | 1.00 (0.60-1.66) | 0.380 | 0.980 |  | 0.578 |
| Current smoker | 0.51 (0.27-0.97) | 0.48 (0.23-1.01) | 0.078 | 0.057 |  | 0.51 (0.24-1.05) | 0.76 (0.43-1.34) | 0.191 | 0.334 |  | 0.993 |
| Above alcohol limits | 2.35 (1.11-4.97) | 2.08 (0.95-4.56) | 0.068 | 0.092 |  | 1.04 (0.59-1.84) | 0.58 (0.30-1.12) | 0.080 | 0.114 |  | 0.124 |
| Low WEMWBS | 0.62 (0.30-1.28) | 0.54 (0.25-1.17) | 0.245 | 0.119 |  | 0.40 (0.18-0.89) | 0.36 (0.17-0.77) | 0.019 | 0.010 |  | 0.060 |
| Self-reported CVD | 0.97 (0.51-1.83) | 0.44 (0.22-0.88) | 0.043 | 0.020 |  | 0.61 (0.32-1.15) | 0.59 (0.31-1.15) | 0.143 | 0.108 |  | 0.407 |

CI, confidence interval, CVD, cardiovascular disease; IPAQ, Short-form International Physical Activity Questionnaire; MVPA, moderate-to-vigorous physical activity; PASBAQ, Physical Activity and Sedentary Behaviour Assessment Questionnaire; WEMWBS Warwick-Edinburgh Mental Well-Being Scale

Estimates age-standardised using the 2012 English household population.

^a^ Odds ratios obtained using logistic regression, with the health outcome as dependent variable and tertiles of MVPA as a categorical variable (lowest group as the reference).

^b^ *P-*value for trend obtained using logistic regression, with the health outcome as dependent variable and tertiles of MVPA entered as a single continuous independent variable.

^c^ PASBAQ- and IPAQ-MVPA included in the same model (adjusted for age) as continuous independent variables; *P*-value shown is the test for statistical interaction.
