# Supplementary material for: Comparison of the Physical Activity and Sedentary Behaviour Assessment Questionnaire and the Short-Form International Physical Activity Questionnaire: An Analysis of Health Survey for England Data
Source: PLoS One. 2016 Mar 18;11(3):e0151647. doi: 10.1371/journal.pone.0151647 (PMC4798726; doi:10.1371/journal.pone.0151647)
Supplement: S4 Table — (DOCX) [file pone.0151647.s006.docx]

**S4 Table** Kappa statistic and 95% CI, and the prevalence-adjusted bias-adjusted Kappa (PABAK) statistic for PASBAQ- and IPAQ-based estimates of sufficient aerobic activity and inactivity excluding occupational activity from MVPA.

|  | **Sufficient activity^a^**  **(MVPA ≥150minutes/weekday)** | | | | | **Inactivity^a^**  **(MVPA <30minutes/weekday)** | | | | |
| --- | --- | --- | --- | --- | --- | --- | --- | --- | --- | --- |
|  | **Kappa**  **(95% CI)** | **K_max_** | **PABAK** | **PI** | **BI** | **Kappa**  **(95% CI)** | **K_max_** | **PABAK** | **PI** | **BI** |
| **All** | 0.27 (0.21-0.33) | 0.60 | 0.36 | -0.38 | 0.18 | 0.24 (0.17-0.30) | 0.62 | 0.59 | 0.68 | -0.10 |
| **Sex:** |  |  |  |  |  |  |  |  |  |  |
| Men | 0.32 (0.24-0.41) | 0.70 | 0.49 | -0.51 | 0.11 | 0.27 (0.17-0.37) | 0.64 | 0.63 | 0.71 | -0.09 |
| Women | 0.29 (0.22-0.36) | 0.59 | 0.33 | -0.32 | 0.19 | 0.21 (0.12-0.29) | 0.59 | 0.54 | 0.66 | -0.12 |
| **Age-group:** |  |  |  |  |  |  |  |  |  |  |
| 16-44 | 0.20 (0.10-0.30) | 0.54 | 0.41 | -0.54 | 0.17 | 0.02 (0.00-0.10) | 0.40 | 0.69 | 0.83 | -0.09 |
| 45-64 | 0.25 (0.16-0.34) | 0.62 | 0.30 | -0.32 | 0.18 | 0.24 (0.13-0.35) | 0.68 | 0.55 | 0.65 | -0.09 |
| 65+ | 0.33 (0.24-0.43) | 0.62 | 0.32 | -0.13 | 0.20 | 0.31 (0.20-0.41) | 0.67 | 0.41 | 0.40 | -0.14 |
| **BMI group:** |  |  |  |  |  |  |  |  |  |  |
| Normal | 0.27 (0.17-0.38) | 0.58 | 0.42 | -0.48 | 0.17 | 0.20 (0.07-0.33) | 0.56 | 0.70 | 0.80 | -0.08 |
| Overweight | 0.20 (0.11-0.29) | 0.53 | 0.33 | -0.44 | 0.20 | 0.14 (0.04-0.24) | 0.59 | 0.56 | 0.71 | -0.10 |
| Obese | 0.23 (0.12-0.34) | 0.64 | 0.27 | -0.27 | 0.17 | 0.20 (0.07-0.33) | 0.67 | 0.47 | 0.60 | -0.11 |
| **Income:** |  |  |  |  |  |  |  |  |  |  |
| Highest | 0.15 (0.04-0.26) | 0.69 | 0.37 | -0.52 | 0.11 | 0.13 (0.01-0.26) | 0.60 | 0.68 | 0.80 | -0.07 |
| Middle | 0.25 (0.15-0.34) | 0.55 | 0.31 | -0.36 | 0.20 | 0.17 (0.05-0.29) | 0.61 | 0.55 | 0.69 | -0.11 |
| Lowest | 0.37 (0.26-0.47) | 0.60 | 0.41 | -0.33 | 0.19 | 0.40 (0.28-0.52) | 0.63 | 0.62 | 0.62 | -0.12 |
| **Heart rate:** |  |  |  |  |  |  |  |  |  |  |
| Lowest | 0.23 (0.12-0.34) | 0.63 | 0.37 | -0.45 | 0.15 | 0.13 (0.00-0.26) | 0.60 | 0.64 | 0.77 | -0.09 |
| Middle | 0.23 (0.13-0.34) | 0.53 | 0.33 | -0.40 | 0.21 | 0.23 (0.11-0.36) | 0.60 | 0.63 | 0.72 | -0.10 |
| Highest | 0.35 (0.25-0.45) | 0.61 | 0.37 | -0.26 | 0.19 | 0.28 (0.17-0.39) | 0.60 | 0.46 | 0.53 | -0.15 |

BI, bias-index; BMI, body mass index; CI, confidence interval; IPAQ, Short-form International Physical Activity Questionnaire; κ_max_, maximum attainable value of the Kappa statistic; MVPA, moderate-to-vigorous physical activity; PABAK, prevalence-adjusted bias-adjusted Kappa statistic; PASBAQ, physical activity and sedentary behaviour questionnaire; PI, prevalence-index.

Bias-index denotes the difference between disagreements; Prevalence-index denotes the difference between agreements on the positive and negative classification.

^a^ PASBAQ-defined sufficient aerobic activity/inactivity included walking of at least moderate-intensity only; IPAQ-defined sufficient aerobic activity/inactivity included all walking as intensity of walking not assessed.
