# Supplementary material for: Comparison of the Physical Activity and Sedentary Behaviour Assessment Questionnaire and the Short-Form International Physical Activity Questionnaire: An Analysis of Health Survey for England Data
Source: PLoS One. 2016 Mar 18;11(3):e0151647. doi: 10.1371/journal.pone.0151647 (PMC4798726; doi:10.1371/journal.pone.0151647)
Supplement: S5 Table — Estimates of MVPA excluded occupational physical activity. (DOCX) [file pone.0151647.s007.docx]

**S5 Table** Associations of PASBAQ- and IPAQ-assessed time spent in MVPA with odds of unfavourable health outcomes. Estimates of MVPA excluded occupational physical activity.

| **Health outcomes** | **PASBAQ-MVPA** | | | |  | **IPAQ-MVPA** | | | |  | **PASBAQ and IPAQ** |
| --- | --- | --- | --- | --- | --- | --- | --- | --- | --- | --- | --- |
|  | **Middle** | **Highest** | *P*^a^ | *P*^b^ |  | **Middle** | **Highest** | *P*^a^ | *P*^b^ |  | *P*^c^ |
|  | **Odds ratio (95% CI)** | |  |  |  | **Odds ratio (95% CI)** | |  |  |  |  |
| **Men** |  |  |  |  |  |  |  |  |  |  |  |
| Raised cholesterol | 0.82 (0.39-1.73) | 0.86 (0.45-1.63) | 0.859 | 0.675 |  | 0.57 (0.32-1.00) | 0.63 (0.37-1.10) | 0.112 | 0.151 |  | 0.315 |
| Obese | 0.86 (0.44-1.67) | 0.87 (0.47-1.60) | 0.870 | 0.679 |  | 0.42 (0.20-0.88) | 0.58 (0.27-1.26) | 0.067 | 0.217 |  | 0.996 |
| Hypertension | 0.74 (0.39-1.40) | 0.93 (0.53-1.65) | 0.610 | 0.910 |  | 1.07 (0.59-1.95) | 0.88 (0.47-1.64) | 0.765 | 0.667 |  | 0.366 |
| Current smoker | 0.79 (0.38-1.67) | 0.57 (0.27-1.19) | 0.282 | 0.122 |  | 1.43 (0.75-2.71) | 1.48 (0.80-2.74) | 0.423 | 0.216 |  | 0.357 |
| Above alcohol limits | 1.87 (0.80-4.39) | 1.19 (0.59-2.41) | 0.283 | 0.804 |  | 0.72 (0.29-1.82) | 0.88 (0.34-2.30) | 0.687 | 0.817 |  | 0.030 |
| Low WEMWBS | 0.40 (0.17-0.95) | 0.26 (0.10-0.69) | 0.019 | 0.009 |  | 0.33 (0.13-0.82) | 0.67 (0.28-1.59) | 0.059 | 0.433 |  | 0.534 |
| Self-reported CVD | 1.13 (0.61-2.09) | 0.69 (0.35-1.38) | 0.435 | 0.245 |  | 0.71 (0.36-1.39) | 0.55 (0.28-1.10) | 0.227 | 0.090 |  | 0.100 |
| **Women** |  |  |  |  |  |  |  |  |  |  |  |
| Raised cholesterol | 1.12 (0.61-2.03) | 0.99 (0.56-1.76) | 0.902 | 0.959 |  | 1.41 (0.80-2.47) | 1.10 (0.60-2.02) | 0.392 | 0.789 |  | 0.715 |
| Obese | 1.23 (0.76-1.98) | 0.52 (0.31-0.87) | 0.004 | 0.010 |  | 0.55 (0.34-0.90) | 0.61 (0.38-0.97) | 0.042 | 0.042 |  | 0.430 |
| Hypertension | 1.66 (1.08-2.57) | 0.83 (0.45-1.51) | 0.016 | 0.491 |  | 0.80 (0.47-1.37) | 0.98 (0.61-1.59) | 0.671 | 0.967 |  | 0.488 |
| Current smoker | 0.46 (0.23-0.95) | 0.81 (0.41-1.58) | 0.093 | 0.565 |  | 0.57 (0.31-1.08) | 1.11 (0.58-2.13) | 0.074 | 0.716 |  | 0.918 |
| Above alcohol limits | 1.68 (0.91-3.09) | 1.57 (0.85-2.93) | 0.211 | 0.160 |  | 1.15 (0.58-2.30) | 0.81 (0.42-1.58) | 0.430 | 0.518 |  | 0.640 |
| Low WEMWBS | 0.66 (0.33-1.31) | 0.41 (0.21-0.80) | 0.035 | 0.011 |  | 0.55 (0.29-1.02) | 0.45 (0.25-0.83) | 0.028 | 0.012 |  | 0.385 |
| Self-reported CVD | 0.67 (0.39-1.17) | 0.38 (0.15-0.94) | 0.097 | 0.027 |  | 0.59 (0.30-1.17) | 0.73 (0.39-1.38) | 0.297 | 0.349 |  | 0.400 |

CI, confidence interval, CVD, cardiovascular disease; IPAQ, Short-form International Physical Activity Questionnaire; MVPA, moderate-to-vigorous physical activity; PASBAQ, Physical Activity and Sedentary Behaviour Assessment Questionnaire; WEMWBS Warwick-Edinburgh Mental Well-Being Scale.

Estimates age-standardised using the 2012 English household population.

^a^ Odds ratios obtained using logistic regression, with the health outcome as dependent variable and tertiles of MVPA as a categorical variable (lowest group as the reference).

^b^ *P-*value for trend obtained using logistic regression, with the health outcome as dependent variable and tertiles of MVPA entered as a single continuous independent variable.

^c^ PASBAQ- and IPAQ-MVPA included in the same model (adjusted for age) as continuous independent variables; *P*-value shown is the test for statistical interaction.
